# A new identified suppressor of Cdc7p/SepH kinase, PomA, regulates fungal asexual reproduction via affecting phosphorylation of MAPK-HogA

**DOI:** 10.1371/journal.pgen.1008206

**Published:** 2019-06-13

**Authors:** Xiaogang Zhou, Jing Ye, Likun Zheng, Ping Jiang, Ling Lu

**Affiliations:** Jiangsu Key Laboratory for Microbes and Functional Genomics, College of Life Sciences, Nanjing Normal University, Nanjing, China; University of Georgia, UNITED STATES

## Abstract

The septation initiation network (SIN), composed of a conserved SepH (Cdc7p) kinase cascade, plays an essential role in fungal cytokinesis/septation and conidiation for asexual reproduction, while the mitogen-activated protein kinase (MAPK) pathway depends on successive signaling cascade phosphorylation to sense and respond to stress and environmental factors. In this study, a SepH suppressor–PomA in the filamentous fungus *A*. *nidulans* is identified as a negative regulator of septation and conidiation such that the *pomA* mutant is able to cure defects of *sepH1* in septation and conidiation and overexpression of *pomA* remarkably suppresses septation. Under the normal cultural condition, SepH positively regulates the phosphorylation of MAPK-HogA, while PomA reversely affects this process. In the absence of PbsB (MAPKK, a putative upstream member of HogA), PomA and SepH are unable to affect the phosphorylation level of HogA. Under the osmostress condition, the induced phosphorylated HogA is capable of bypassing the requirement of SepH, a key player for early events during cytokinesis but not for MobA/SidB, the last one in the core SIN protein kinase cascade, indicating the osmotic stimuli-induced septation is capable of bypassing requirement of SepH but unable to bypass the whole SIN requirement. Findings demonstrate that crosstalk exists between the SIN and MAPK pathways. PomA and SepH indirectly regulate HogA phosphorylation through affecting HogA-P upstream kinases.

## Author summary

Timely septation and conidiation are essential for fungal asexual reproduction. Here, we identified a putative dual-specificity tyrosine phosphorylation-regulated kinase PomA, as a suppressor of a conserved SepH (Cdc7p) kinase in the septation initiation network (SIN) cascade, is a new identified negative regulator for septation and conidiation in *A*. *nidulans*. Our findings indicated that reversible protein phosphorylation of the high-osmolarity glycerol gene *hogA* regulated by the SIN-SepH-PomA kinase cascade plays a key role for fungal cell septation and asexual reproduction. However, when fungi meet osmostress, they bypass the originally required SIN protein cascade to fit the environmental niches by increasing the phosphorylation of HogA.

## Introduction

The filamentous fungus contains a mycelium of multinucleated cells that are partitioned by septa, and timely cytokinesis and septation is essential for hyphal growth and conidiation [1,2]. Conidia are the primary means of asexual reproduction and dispersal in the environment for a variety of fungi [3–5]. Generally, fungal cells respond to environmental stimuli through signaling pathways often involving kinase cascades that transmit external cellular signals to the nucleus [6–8]. Genetic analyses have reported that the model filamentous fungus *Aspergillus nidulans* and *Schizosaccharomyces pombe* have components of the septum initiation network (SIN), and septum formation requires the assembly of a septal band composed of a dynamic protein complex that is dependent upon conserved core SIN components including three protein kinases (SepH/Cdc7p, SidA/Sid1p and SidB/Sid2p), and their regulatory partners (SpgA/Spg1p, Cdc14p and MobA/Mob1p), respectively [2,9,10]. Notably, downregulation of the serine/threonine protein kinase SepH cascade in *A*. *nidulans* would abolish septation and conidiation, its hyperactivation of it would induce the formation of multiple septa. Thus, SepH is a positive regulator of the SIN that triggers septation/conidiation in *A*. *nidulans* [9]. However, whether negative regulators exist for conidiation is elusive. In addition, individual cell signaling pathways can communicate with each other, thereby building a complex network to ultimately control gene expression and other cellular functions [11]. In all eukaryotic kingdoms, mitogen-activated protein kinases (MAPKs) play critical roles in cellular responses to environmental cues [12]. These MAPKs are activated by phosphorylation at highly conserved threonine and tyrosine residues [13] in response to specific inputs, leading to their accumulation in the nucleus and the activation of their downstream targets [14]. Three-tiered core signaling modules, including MAPK kinase kinases (MAPKKK), MAPK kinases (MAPKK) and MAPK, are included in the MAPK signaling pathway [12,15]. Cells respond to different external environment stresses, including pH, light signaling, and osmotic or oxidative stress, by phosphorylating the kinase cascade to activate the MAPK signaling pathway [11,14,16,17]. Thereafter, MAPK is phosphorylated and shuttles into nuclei upon stimuli to trigger gene expression. Thus, reversible protein phosphorylation plays a critical role in the regulation of virtually all eukaryotic biological processes, and the MAPK signaling pathway plays a crucial role in different organisms from mammals to fungi. In mammalian cells, the MAPK signaling pathway is critical for normal immune and inflammatory responses and contributes to cell cycle or cytoskeleton remodeling [18]. In model yeasts, the MAPK signaling pathway involves many biological processes, including cell wall integrity, cell separation and the regulation of mitotic spindle disassembly [15,19]. In most filamentous fungi, the high-osmolarity glycerol (HOG) gene *hogA*, which encodes a MAPK that sense osmotic and oxidative stress, is required for hyphal growth and conidiation under osmotic stress culture conditions [14,20]. Moreover, in the opportunistic fungal pathogen *A*. *fumigatus*, the high-osmolarity glycerol (HOG) MAPK signaling pathway is crucial for virulence and conidiation under stress conditions [12,21]. Therefore, both the SepH-SIN kinase cascade and the HOG-MAPK pathway are involved in cell growth and reproduction by regulating reversible protein phosphorylation. However, whether crosstalk between the SIN cascade and the HOG-MAPK pathway exists remains unknown.

Through UV mutagenesis, our previous studies identified antagonizing components of the SepH kinase cascade [22]. However, this complex protein cascade has not yet been dissected since the majority of mutants isolated by UV mutagenesis have no clear phenotype. Therefore, it is difficult to confirm these related mutations in the complementation assay by transforming a whole-genome library since a critical barrier in forward genetics is to verify gene functions with no detectable phenotypes. With the development of next-generation sequencing (NGS), it is possible to identify these mutations by comparing single nucleotide polymorphisms (SNPs) between mutants and wild type which all have the same parental genetic background. Based on these approaches, in this study, through backcross techniques combined with a new mutation comparison analysis of NGS, a putative protein kinase, PomA, a suppressor of SepH, was identified to be involved in septation and conidiation. Moreover, quantitative phosphoproteomics revealed that deletion of *pomA* causes activation of the HOG MAPK signaling pathway.

## Results

### Screening and isolation of the *sepH1* suppressors of septation and conidiation

**Fig 1:**
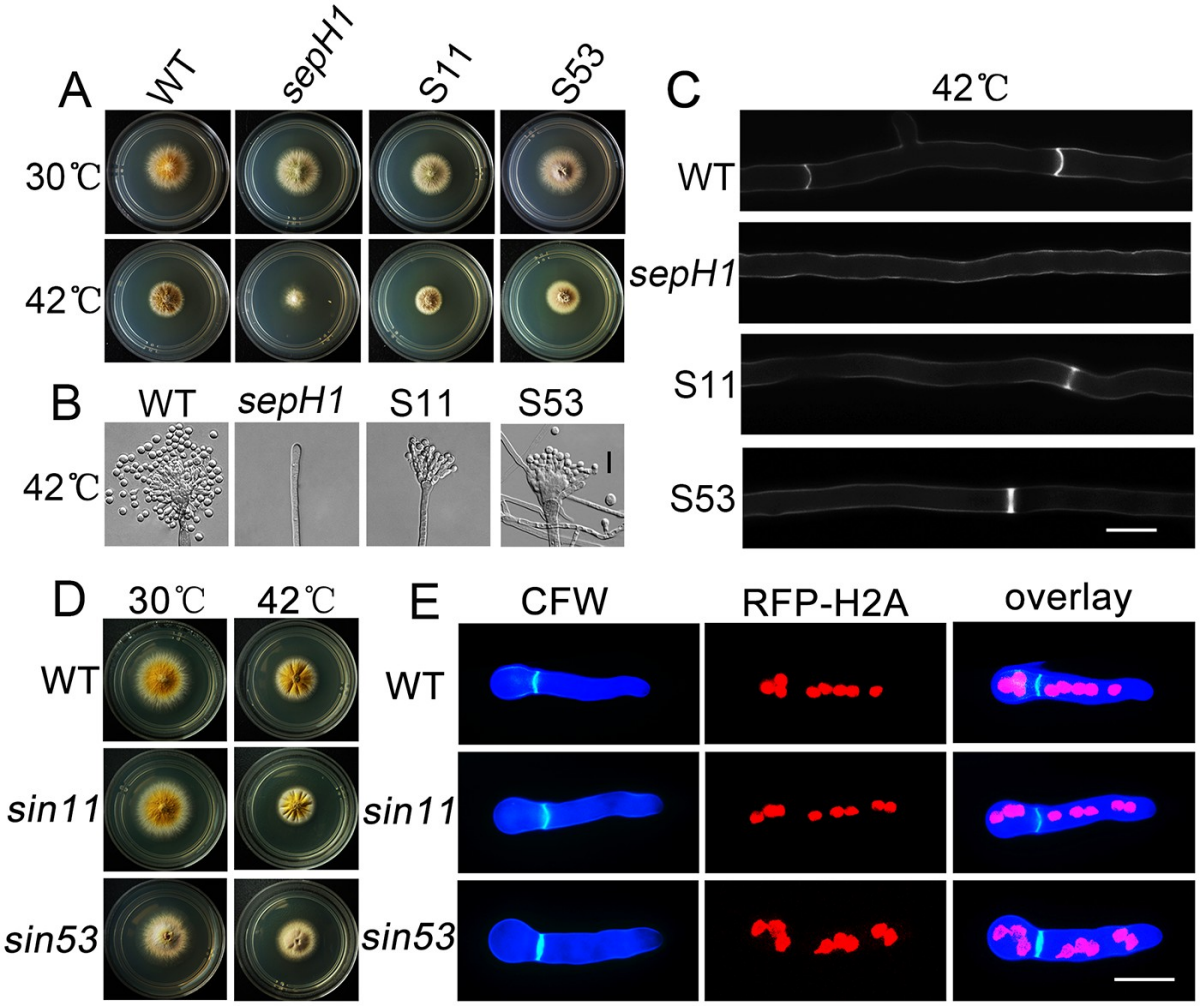
Septation and conidiation were partly rescued in S11 and S53 compared to the *sepH1* mutant at 42 °C. (A) Colony morphologies of the wild type (R21), *sepH1*, S11 and S53 strains cultured on YUU at 30 or 42 °C for 2 days. (B) Conidiophores for the conidia of the wild type (R21), *sepH1*, S11 and S53 strains. Bars, 10 μm. (C) Comparison of septum formation in hyphal cells of the wild type (R21), *sepH1*, S11 and S53 strains cultured on liquid medium YUU at 42 °C for 20 h. Bars, 10 μm. (D) Colony morphologies of the wild type (R21), *sin11* and *sin53* strains cultured on YUU at 30 or 42 °C for 2 days. (E) Morphological comparison of hyphal cells labeled with an RFP-histone H2A tag showing nuclei distribution and stained with CFW showing septa in relative strains cultured in liquid medium YUU at 42 °C for 7.5 h. Bars, 10 μm.

Our previous study suggested three categories among the 116 independent UV-induced suppressor mutations of *sepH1* based on colony phenotype [22]. To further dissect these suppressor-related mutations, we backcrossed these conidiation-restored mutants with the wild type strain R21, which has a yellow colony selection marker (*yA1*). Consequently, the isolated progenies from mutants S11 and S53 (suppressor of *sepH1*, No. 11 and No. 53, respectively) crossed with wild type R21 clearly displayed the predicted phenotypic separation as shown in S1 Fig. The ratio of the four different types of progeny colonies (n = 193 for S11 and n = 218 for S53) was approximately 1:1:1:1 for which genotypes were supposed to be wild type, *sepH1* (referred to as *sepH1-1* and *sepH1-3*, respectively), single mutations No. 11 and No. 53 (referred to as *sin11* and *sin53*, respectively) and double mutations *sepH1*, *sin11* or *sin53* (S2 Fig). To further confirm whether *sin11* and *sin53* truly act as suppressors of *sepH1* on conidiation and septation, we further crossed the isolated *sin11* and *sin53* mutants with *sepH1*. Consequently, four different colony phenotypes were observed again at 30 °C and 42 °C, suggesting that progenies may include four different genotypes: *sin11* or *sin53* and *sepH1*, wild type strains and the putative double mutants *sepH sin11* and *sepH sin53* (S2 Fig). These data suggest that *sin11* and *sin53* are extra-mutations from *sepH1*. Thus, we next selected only S11 and S53 to further examine the phenotypes. As shown in S3A Fig, the production of conidia and the development of conidiophores were rescued to some extent in mutants S11 and S53 compared to that of the *sepH1* mutant with a completely abolished conidiophore structure under the same cultural restriction temperature (42 °C) (Fig 1A and 1B). Consistently, liquid culture experiments demonstrated that both S11 and S53 displayed calcofluor white (CFW)-stained septa in hyphal cells compared to *sepH1* with no detectable sign of the septum in hyphal cells at 42 °C (Fig 1C and S3B Fig), suggesting that defects of septation in *sepH1* at 42 °C could be suppressed by mutation in S11 or S53. To further verify the phenotypes from isolated *sepH1* progenies, hyphal cells of *sepH1-1* and *sepH1-3* were stained with CFW. No stained septa were observed in *sepH1-1* or *sepH1-3*, while double mutants *sepH sin11-2* and *sepH sin53-2* displayed CFW-stained septa in hyphal cells at 42 °C (S2C Fig), suggesting that mutations *sin11* and *sin53* truly suppress the defects of *sepH1* on septation. In addition, strains *sin11* had slightly reduced colony sizes compared to the wild type strain R21 at 42 °C (Fig 1D and S3D Fig). Quantification of septa, conidia and colony diameters for indicated strains as shown in S3 Fig. To further visualize the cellular phenotype caused by mutations *sin11* and *sin53*, a red fluorescent protein (RFP) tag was tagged to the N-terminus of histone H2A, a nuclear protein marker, in the respective background of wild type, *sin11* and *sin53*; the resulting strains were named ZXA05, ZXA06 and ZXA07. Distribution of septa stained by CFW and nuclei fused RFP with a nuclei marker histone H2A were displayed in related strains. Fluorescence microscopy revealed no detectable difference in the distribution of nuclei or septa, even for hyphal polarity growth in the isolated *sin11* and *sin53* mutants (Fig 1E). Therefore, these data collectively imply that the mutation *sin11* or *sin53* may not cause the remarkable growth phenotype (only with slightly slow colony growth), but is able to suppress defects of *sepH1* function for septation.

### A putative protein kinase, PomA, as a suppressor of SepH, is involved in septation

**Fig 2:**
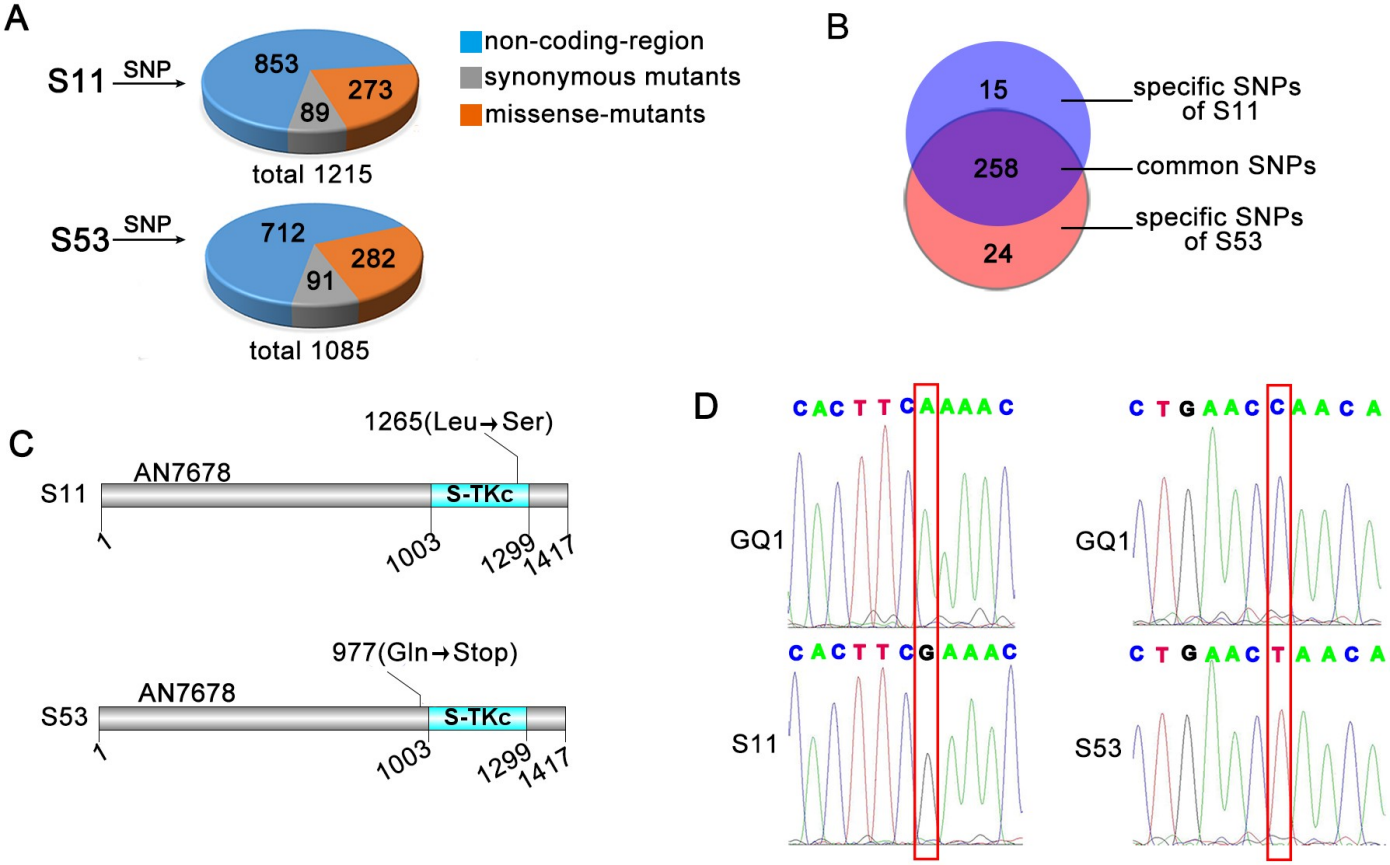
Single nucleotide polymorphism comparison and identification of the *pomA* mutation in S11 and S53. (A) (B) SNP comparison by next-generation sequencing for the whole genomes of S11 and S53. (C) Domain analysis of PomA with SMART (http://smart.embl-heidelberg.de/). (D) Mutations in the cloned *pomA* gene from S11 and S53 identified by sequencing.

**Fig 3:**
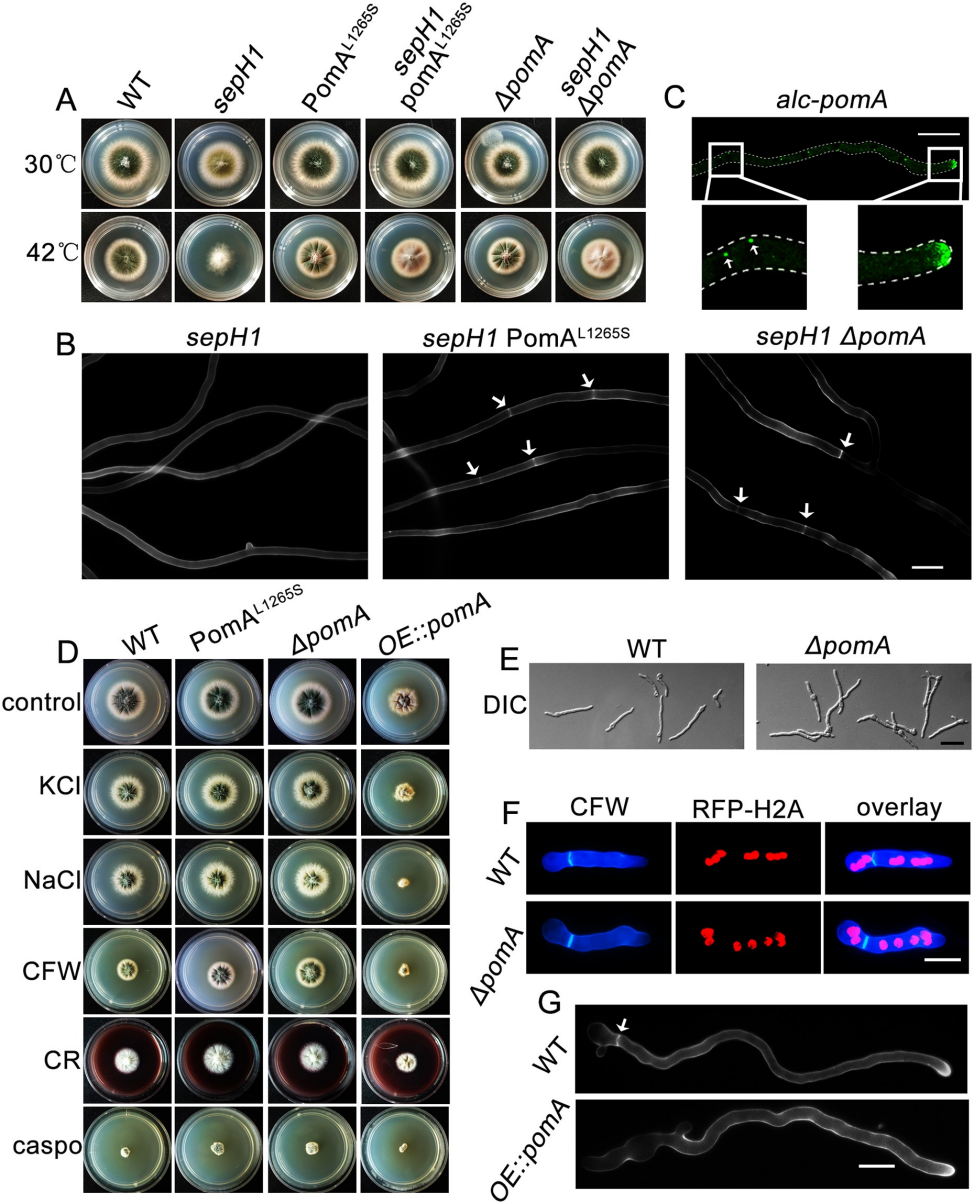
PomA mutation or deletion suppressed *sepH1* defects in septation and conidiation. (A) Colony morphologies of the wild type (TN02A7), *sepH1*, *ΔpomA*, PomA^L1265S^, *sepH1 ΔpomA* and *sepH1* PomA^L1265S^ strains cultured on YUU at 30 or 42 °C for 2 days. (B) Septa comparison in hyphal cells stained with CFW in the indicated strains cultured in liquid rich media YUU at 42 °C for 20 h. Arrows indicate the locations of septa. Bars, 10 μm. (C) Localization of GFP-PomA under control of the *alcA* conditional promoter when strain ZXA14 was cultured with liquid minimal media PGRT. Bars, 10 μm. (D) Colony morphologies of the indicated strains cultured on YAG medium or YAG medium supplemented with 1 M KCl, 1 M NaCl, calcofluor white (CFW) (50 μg/ml), Congo red (CR) (100 μg/ml) and caspofungin (1.25 μg/ml) at 37 °C for 2 days. (E) Germling morphological comparison by differential interference contrast (DIC) images of the parental wild type (TN02A7) and *ΔpomA* strains cultured in liquid media YUU for 8 h. Bars, 10 μm. (F) Morphological comparison of hyphal cells labeled with an RFP-histone H2A tag showing nuclei distribution and strained with CFW showing septa in the related strains cultured in liquid medium YUU at 37 °C for 7 h. Bars, 10 μm. (G) Comparison of formed septa in hyphal cells stained with CFW in the parental wild type (TN02A7) and *OE*::*pomA* strains cultured in liquid media YUU at 37 °C for 10 h. Bars, 10 μm.

To further identify which mutated gene contributes to the suppression of *sepH1* in septation and conidiation, SNP comparison by NGS for the whole genomes of S11 and S53 was carried out. Since mutants S11 and S53 were derived from the same background strain, *sepH1* (compared to the *A*. *nidulan*s A4 genome database), their SNPs should be consistent except for those mutations induced by UV in *sin11* and *sin53*. Total 1215 and 1085 SNPs were found, while 273 and 282 belonged to missense mutations occurring in the coding region in S11 and S53, respectively (Fig 2A and S1 Data File). Given that mutations that contributed to the suppression of *sepH1* in S11 and S53 should differ since they were independently isolated from UV-induced mutants, the overlapping common SNPs were excluded. As a consequence, 15 and 24 specific SNPs remained in S11 and S53, respectively (Fig 2B). After a BLAST analysis of the whole genomic sequence assembly compared to the *A*. *nidulans* FGSC A4 (http://www.aspgd.org/) genome for these specific SNPs in S11 and S53, interestingly, multiple different SNPs were located in three genes (ID numbers: AN7678, AN7949 and AN10000), while AN7678 had a high-quality score. Hence, we next determined whether the AN7678 mutation contributed to the suppression of *sepH1* on septation and conidiation. According to information at FungiDB (http://fungidb.org/fungidb/), the gene AN7678 has 4482 nucleotides and encodes a putative 1417-aa protein. From information obtained from a homology search by Blast-P in the *S*. *pombe* database, AN7678 shares 58% identity to that of Pom1, a DYRK family cell polarity protein kinase in *S*. *pombe*, which, according to SMART analysis (http://smart.embl-heidelberg.de/), harbors a conserved domain, S-TKc (Serine/Threonine protein Kinases, catalytic domain), ranging from 1003 to 1299 aa. Conserved domain comparison of the S-TKc domain between Pom1 in *S*. *pombe* and AN7678 indicated high identity (68%). Hence, these results suggest that AN7678 is a homolog of Pom1 in *A*. *nidulans*; thus, we then named AN7678 as PomA. According to SNP sequencing and alignment information, we found that S11 harbors a mutation in AN7678 in which leucine^1265^ is replaced by serine, which is located in the S-TKc domain. Notably, the mutation occurring in S53 at codon 977 produces a stop codon before the S-TKc domain (Fig 2C). Further cloning and sequencing of the *pomA* gene from mutants S11 and S53 consistently confirmed that the aforementioned mutations truly happened (Fig 2D). To further demonstrate whether septum recovery and the suppression of *sepH1* in S11 and S53 were a specific result of the PomA mutation, we then generated a site-directed mutation strain, PomA^L1265S^, that mimics the mutation in S11 (Leu to Ser at the position 1265) and a full-length *pomA* deletion strain in the background of *sepH1* and its parental wild type strain. As shown in Fig 3A, there were no significant different colony phenotypes in the *pomA* deletion, PomA^L1265S^ mutant compared to that their parental wild type. However, in double mutants *sepH1*, *ΔpomA* and *sepH1* PomA^L1265S^, there were some rescued conidiation on agar-solid media and recovered septation in liquid media (Fig 3A and 3B, S3B Fig), suggesting both the *pomA* null and PomA^L1265S^ site-directed mutants were able to suppress septation and conidiation defects induced by *sepH1* (Fig 3A and 3B). Thus, these data repeatedly demonstrated that double mutations of *sepH* and *pomA* were capable of recovering septation and conidiation.

In addition, to provide further insights into the functions of PomA, a conditional *alcA(p)*::GFP*-pomA* strain was constructed by homologous integration in which the *pomA* gene was under the control of different carbon source de-repression/repression by glycerol/glucose and induction by threonine. GFP-PomA showed a similar pattern of localization with the highly accumulated at the tips of hyphal cells and the analogous spindle pole body near the nucleus when cultured either in de-repression glycerol or induction-threonine media (Fig 3C), suggesting that PomA may be involved in cell division or hyphal cell polarity growth. However, the *ΔpomA* and PomA^L1265S^ mutants showed almost similar colony phenotypes compared to their parental wild type strain regardless of culture conditions (in rich media YAG or supplemented with 1 M NaCl or KCl) (Fig 3D–3F and S4A Fig). In contrast, when cultured in media supplemented with the fungal cell wall-perturbing agent congo red (CR) or CFW, the *pomA* mutants showed more resistance to these reagents than their parental wild type strain to some extent as shown in S4A Fig for quantified data. However, the overexpression of *pomA* in a wild type reference strain (2-fold changes of mRNA shown in S4B Fig) displayed very sick colony phenotypes with no septa stained by CFW in germlings (Fig 3D and 3G and S4A Fig) in all tested culture media. These data suggest that excess PomA was toxic and inhibited cell growth and development, implying that PomA is a negative regulator of these processes.

### Quantitative phosphoproteomics revealed that the deletion of *pomA* causes a highly activated HOG pathway

**Fig 4:**
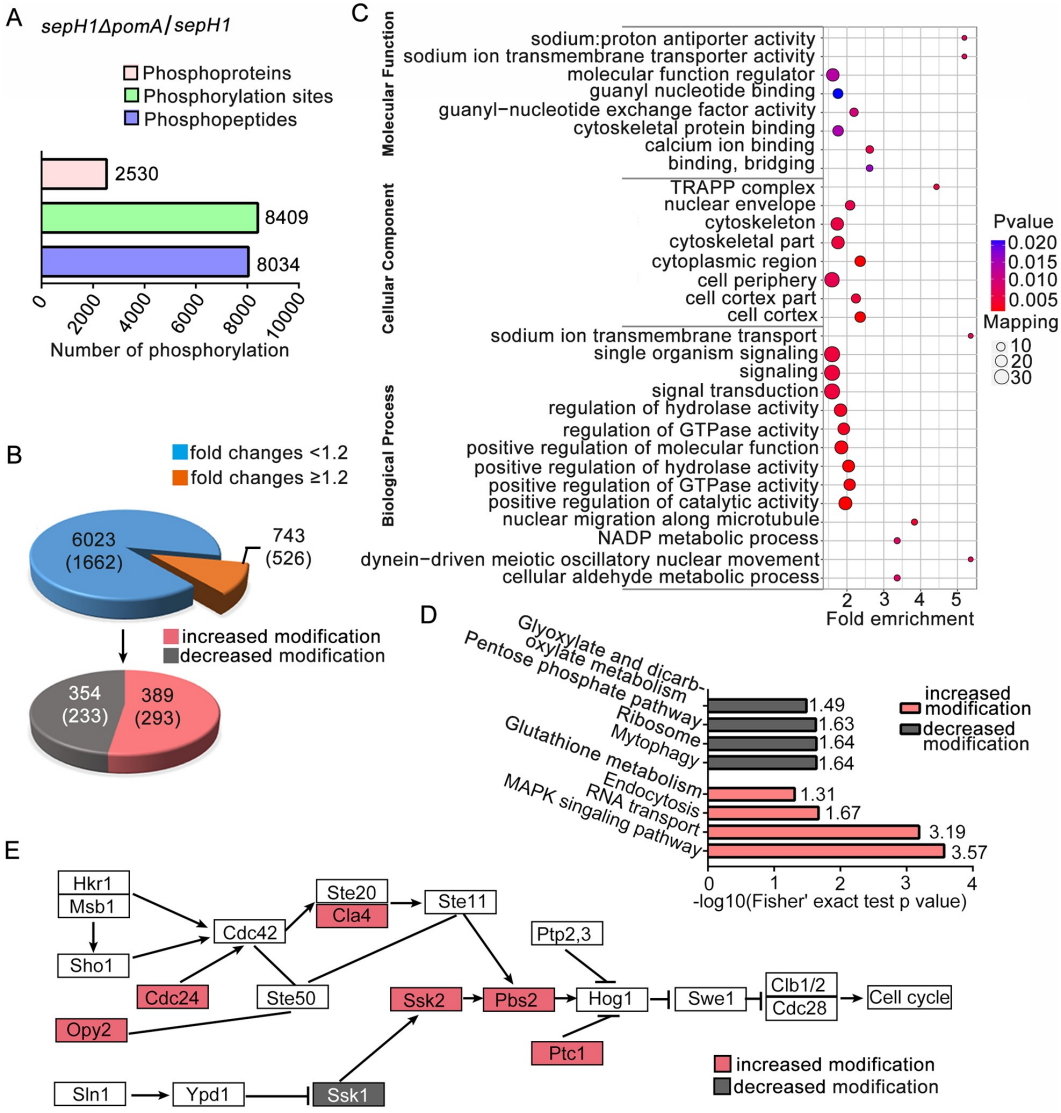
Quantitative phosphoproteomics comparison between the septa-restored strain *sepH1ΔpomA* (ZXA10) and the septa-abolished control strain *sepH1* revealed that the deletion of *pomA* activated the HOG pathway. (A) Total number of detected phosphorylation sites, peptides and proteins and (B) fold changes of phosphorylated proteins. (C) GO terms enriched for all phosphorylated proteins and (D) KEGG pathways enriched in phosphorylated proteins with more than 1.2-fold changes. (E) Visual analysis of the increased phosphorylated proteins in the MAPK pathway (labeled in red).

Considering that homologs of PomA and SepH in yeasts (Pom1 and Cdc7p) belong to the protein kinases that execute major functions by phosphorylating their related substrates, we then carried out tandem mass tag (TMT)-based quantitative phosphoproteomics to analyze the overall phosphorylation levels in the septa-restored strain *sepH1 ΔpomA* (ZXA10) and the septa-abolished control strain *sepH1*. A total of 8409 phosphorylation sites involved in 2530 proteins were identified, in which 6766 phosphorylation sites mapped to 2188 proteins had convincible quantitative detection (Fig 4A and S2 Data File). Using a cut-off was considered to be statistically significant (p-value < 0.05) for 1.2-fold changes in which fold changes has a ratio-compression effect result from isobaric mass tagging techniques for the phosphorylated proteins [23]. Normalized quantitative proteomics revealed 389 (293) increased and 354 (233) decreased phosphorylation modification sites (proteins) in *sepH1 ΔpomA* compared to *sepH1* (Fig 4B). According to GO categories, these phosphorylation modification site-containing proteins are enriched in cellular and metabolic processes (Fig 4C). Moreover, using protein pathway annotation by the Kyoto Encyclopedia of Genes and Genomes (KEGG) database, for which p-values are less than 0.05, these phosphorylation-increased proteins were mainly mapped to four KEGG signaling pathways (Fig 4D). Among them, the HOG MAPK signaling pathway gained the highest score, suggesting that the loss of *pomA* enhances phosphorylation levels in the MAPK pathway. To further verify these phosphorylation-changed proteins-involved in pathways, we re-calculated data in phosphoproteomics based on increased value of cutoff to 1.3-fold. As shown in S5 Fig, these phosphorylation-increased proteins still are mapped to the MAPK signaling pathway, suggesting this pathway may involve in defected *pomA*-induced septation. The quantitative phosphoproteomics was normalized by the proteomics of total proteins while expression of HogA was too low to be considered based on the measurement probably due to strains were cultured under the normal cultural condition. Based on fission yeast *S*. *pombe* MAPK signaling pathway information, further visual analysis revealed six homologous components in the MAPK signaling pathway (labeled as “red” in Fig 4E) with increased phosphorylation levels in the *sepH1 ΔpomA* double mutant compared to the *sepH1* single mutant, indicating that a lack of PomA causes increased phosphorylation of MAPK. Therefore, quantitative phosphoproteomics data imply that PomA works as a negative regulator to regulate the phosphorylation level of MAPK.

### SepH and PomA coordinately regulate the phosphorylation of HogA

**Fig 5:**
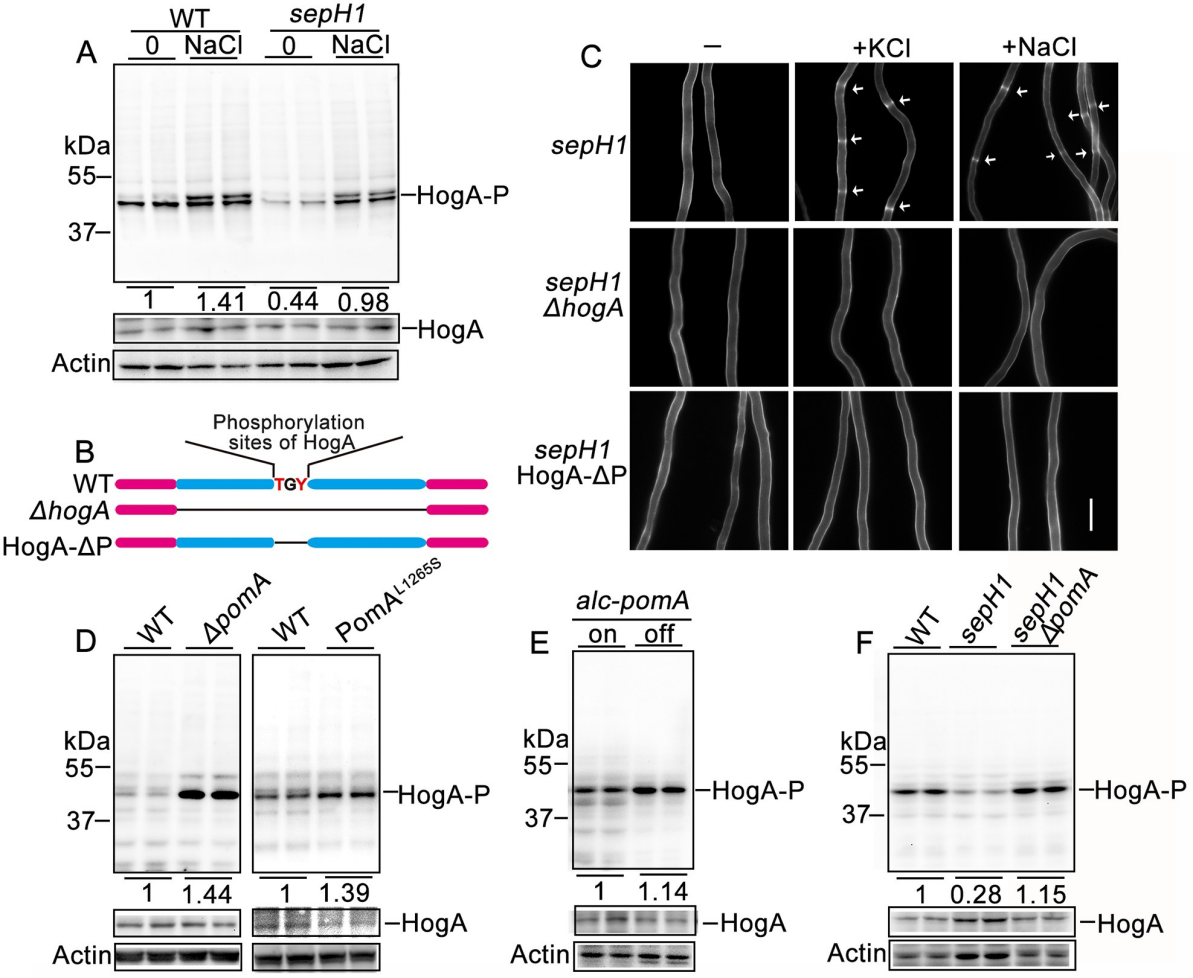
PomA and SepH reversely affected the phosphorylation of HogA. (A) The phosphorylation level of wild type (TN02A7) and *sepH1* strains cultured in liquid minimal media PGRUU treated with or without 1 M NaCl for 10 min at 42 °C for 24 h. (B) Diagram showing strategies of HogA full-length deletion and deleted for predicted phosphorylation sites (171–173) of HogA. (C) Comparison of hyphal cells stained with CFW for the *sepH1*, *sepH1 ΔhogA* and *sepH1* HogA-ΔP with or without treatment of 1 M NaCl or 1 M KCl at 42 °C. Arrows indicate the locations of septa. Bars, 10 μm. (D) Western blot analysis showing the expression level of P-HogA in the *pomA* null mutant and the *pomA* site-directed strain cultured in liquid minimal media PGRUU at 37 °C or (F) sepH1 relative strains at 42 °C for 24 h. (E) Western blot analysis showing the expression level of P-HogA in the *alc(p)*::GFP-*pomA* strain cultured in de-repressed media MMPGR at 37 °C for 24 h or transferred to repressed media YAG for 30 min.

To further determine whether phosphorylation levels in the MAPK pathway could be affected by SepH or PomA, we next verified the phosphorylation status of the MAPK HogA (yeast *S*. *cerevisiae* Hog1 homolog in *Aspergillus*), the final-step kinase in this pathway that is phosphorylated in response to external environment stresses in the activated MAPK cascade. Western blot analysis revealed a clear band of the predicted size in the *A*. *nidulans* wild type strain probed with the antibody against phospho-p38 MAPK (HogA homolog in mammals) (S6 Fig). Upon deleting the *hogA* gene or three key amino acids for predicted phosphonate sites at HogA (Fig 5B), this predicted HogA-P band was completely disappeared, indicating that this antibody might be able to recognize HogA-P (S6 Fig). Therefore, we used this antibody to further analyze the phosphorylation status of HogA induced by the high-osmolarity stimulus, NaCl (1 M). The protein band intensity was remarkably increased in the NaCl-induced sample compared to the control (Fig 5A), suggesting that the phosphorylation level of HogA is truly increased after treatment with NaCl for 10 min, further implying that this antibody is able to recognize HogA phosphorylation. Thus, we used this antibody to determine whether there were any altered phosphorylation levels in the aforementioned *pomA*- and *sepH1*-related mutants. Compared to the parental wild type strain, *sepH1* caused reduced expression of HogA-P while deletion of *pomA* or site-directed PomA^L1265S^ increased HogA-P expression (Fig 5A, 5D and 5F). Notably, in the background of *sepH1*, lack of PomA recovered HogA-P expression to a level similar to that of wild type (Fig 5F). These data suggest that SepH and PomA coordinately regulate the phosphorylation of HogA, likely playing a key role in septation and conidiation. Moreover, to further investigate whether the upstream kinase of HogA is required for phosphorylation of HogA regulated by SepH and PomA, we deleted PbsB (MAPKK, a putative upstream member of HogA) in the background of wild type (TN02A7), *ΔpomA* and *sepH1*, respectively. As shown in S7 Fig, all *ΔpbsB* related strains showed no detectable band of HogA-P in the Western blotting compared to its parental wild type with a predicted HogA-P band, suggesting PbsB is required for phosphorylation of HogA. In the absence of PbsB, PomA and SepH were unable to affect the phosphorylation level of HogA which suggest that PomA and SepH indirectly regulate HogA phosphorylation may via affecting HogA-P upstream kinases. Meanwhile, it also suggests that the antibody could recognize HogA-P. We next tested whether the increased expression of HogA-P induced by osmotic stress could bypass the requirement of SepH. Hyphal cells of the *sepH1* mutant cultured in liquid MM medium with NaCl or KCl were stained with CFW, which clearly showed that the *sepH1* mutant was able to produce septa culturing at 42 °C (Fig 5C), suggesting that osmotic stress bypasses the requirement of *sepH1*. This result also suggests that septation defects in *sepH1* may be due to the reduced expression of HogA-P. These phenomena also coincided with the expression level of HogA-P in the *sepH1* mutant, suggesting that the phosphorylated activation of MAPK may play important roles in septation and conidiation. In contrast, the deletion or turn off of *pomA* expression in a conditional strain enhanced the expression of HogA-P (Fig 5D and 5E), demonstrating that PomA works as a negative regulator for the phosphorylation of HogA. We next deleted *hogA* gene or three key amino acids for phosphonate sites at HogA in the background of *sepH1* to obtain a double-mutation strain *hogA*, *sepH1* for which osmotic stresses were no longer able to induce septation again, further demonstrating HogA is required for bypassing *sepH1* to respond stress stimuli for septation (Fig 5C). Moreover, to gain insights in the actual cross-talk point between the HOG and the SIN, we carried out a strains-crossing technique to make a conditional stain *alc (p)*:: GFP-*mobA*, *sepH1* in which MobA is a partner of SidB, a downstream kinase of SepH in the SIN pathway. In this strain, MobA was conditional expressed and labelled by GFP. As shown in S8A and S8D Fig, turn-off of *mobA* was no longer to induce septation by the osmotic stress stimulus although NaCl addition still could induce enhanced expression of HogA-P. Comparably, deletion of a *mobA* paternal, *sidB*, also was unable to induce septation (S8B Fig). These data collectively suggest that complex MobA/SidB, downstream targets of SIN-SepH, is required for septation even under the osmotic-stress condition. Moreover, in the background of *sepH1*, we found that GFP-MobA from observation for localization, could not translocate to the septum site instead still localized at the site of the spindle pole body no matter under the normal or the osmotic stress culture condition (S8E Fig). In addition, deletion of *pomA* was unable to rescue septation when turn-off expression of MobA as shown in S8C Fig. These data imply that PomA is a suppressor of SepH but not for MobA/SidB and SepH is required for the MobA translocation to septa. Thus, the osmotic stimuli-induced septation is capable of bypassing requirement of SepH but unable to bypass the whole SIN requirement.

### AnkA, a yeast *S*. *pombe wee1* homolog in *A*. *nidulans* has a similar suppression function with PomA against SepH

**Fig 6:**
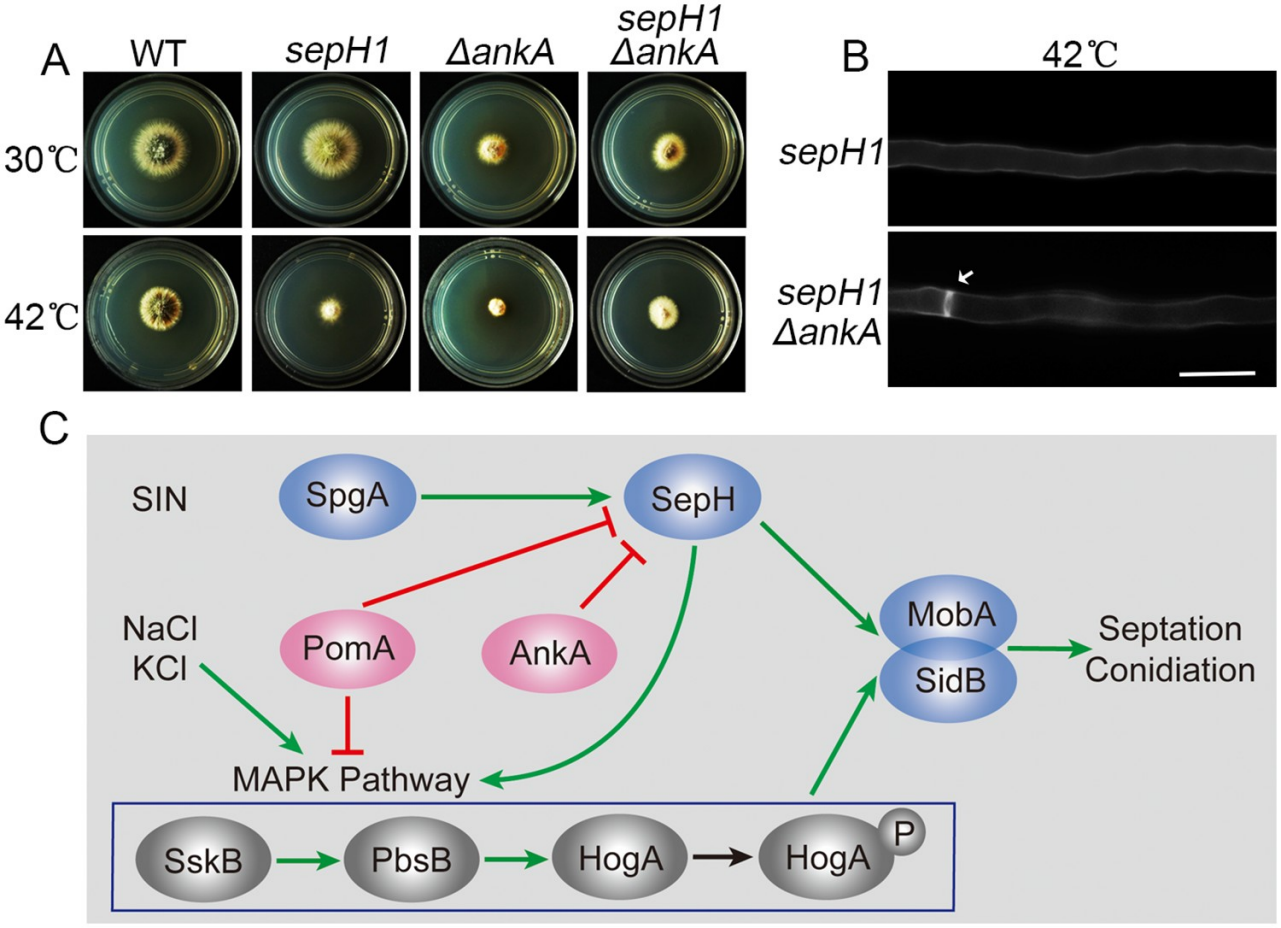
Suppressed *sepH1* defects by deletion AnkA in septation and a cross-link working model between the SIN and the MAPK. (A) Colony morphologies of the wild type (TN02A7), *sepH1*, *ΔankA sepH1* and *ΔankA* strains cultured on YUU at 30 or 42 °C for 2 days. (B) Septa comparison in hyphal cells stained with CFW in the indicated strains cultured in liquid rich media YUU at 42 °C for 20 h. Arrows indicate the locations of septa. Bars, 10 μm. (C) A schematic model for the MAPK and the SIN-SepH-MobA/SidB pathways regulated by PomA-AnkA regulation during septation and conidiation. Green arrows indicate induce and labels for red lines indicate inhibit.

In fission yeast *S*. *pombe*, Pom1 regulates the cell cycle by modulating the activity of a Pom1/Cdr2/Wee1 geometry network in which Pom1 negatively regulates Cdr2, a previously described Wee1 inhibitor, suggesting that Pom1 and Wee1 play a similar role in the cell cycle [24–27]. To address whether *ankA* has suppression function to *sepH1* in *A*. *nidulans*, we deleted *ankA*, a *wee1* homolog in *A*. *nidulans* [28,29], in the background of *sepH1*, which was named ZXA17 (Fig 6A). Consequently, *ΔankA sepH1* exhibited rescued septation accompanied with more robust colony growth and abundant conidia production than that of the *sepH1* mutant (Fig 6A) and rescued septation (Fig 6B and S3B Fig) under the condition of a restrictive temperature (42 °C), suggesting that *ankA* is able to suppress the *sepH1* defects in septation and conidiation, consistent with that of *pomA*. Therefore, these data suggest that *A*. *nidulans* may also have a PomA/Cdr2homolog/AnkA (Wee1) geometry network that is similar to that of *S*. *pombe* Pom1/Cdr2/Wee1. Taken together, PomA suppresses the defects caused by the loss of function of SepH, which may regulate MAPK activity via the PomA/AnkA network to suppress the defects of SepH (Fig 6C).

## Discussion

In this study, by SNPs comparison and the backcross techniques between the mutant and the same parental background wild-type strain, we isolated a new suppressor of SepH (an SIN cascade component), PomA. Our principal conclusions are as follows. First, a putative protein kinase, PomA, in *A*. *nidulans* localized with a spindle pole body-like pattern and the tips of hyphal cells, as a suppressor of SepH, is involved in septation and conidiation such that lack of PomA cures defects of septation and conidiation in *sepH1* while overexpression of PomA remarkably abolishes septation and condiation. Second, SepH positively regulates the phosphorylation of HogA, while PomA reversely affects this process. Third, the phosphorylation of HogA induced by osmotic stress bypasses the requirement of SepH to fulfill septation and conidiation. We demonstrated that crosstalk exists between the SIN and MAPK pathways such that PomA and SepH indirectly regulate HogA phosphorylation through affecting HogA-P upstream kinases.

Several lines of evidence indicate that SepH, a homolog of serine-threonine kinase Cdc7p in fission yeast, is a main positive regulator in the SIN in *A*. *nidulans* [9,30,31]. The *sepH1* mutant with completely abolished septation and fluffy colony phenotypes was previously isolated by screening in temperature-sensitive cytokinesis mutants [9,22,31]. Pom1, a dual-specificity tyrosine phosphorylation-regulated kinase in *S*. *pombe* [32], inhibits the SAD-like kinase Cdr2 and Mid1/anillin to negatively control cell division plane positioning and restricts the cell middle cortical cytokinetic ring precursor nodes through an unknown mechanism [32–35]. The Pom1 mutants divide at a significantly shorter size [32] than wild type cells and some mutants display an abnormal growth axis, resulting in angled and branched cells [36]. These findings suggest that Pom1 in *S*. *pombe* is required for the timing of cytokinesis and for normal polarized cell growth [32,34,36–39]. However, the functions of the Pom1 homolog PomA in *A*. *nidulans* have not yet been reported. Our findings suggest that the loss of *pomA* causes no detectable difference in hyphal cell polarized growth or in septation compared with the wild type strain, even though PomA contains the SPB and exhibits cell tip localization patterns that are similar to those of Pom1 (Fig 3C–3F). This phenomenon may also suggest other alternative candidates to bypass the requirement for polarity growth and for septation in the absence of PomA. In contrast, the overexpression of *pomA* caused significantly reduced colony growth accompanied with abolished septation/conidiation (Fig 3D and 3G), suggesting that PomA is a negative regulator of this process and also implying PomA is a potential efficient inhibitor for conidiation. Our findings demonstrated that osmotic stressors NaCl or KCl were able to induce septation and conidiation regardless of the absence or presence of the SIN component SepH (Fig 5C). These data indicate that when fungi sense environmental stresses, they bypass the originally required SIN protein cascade-SepH to fit the environmental niches by highly expressing phosphorylated HogA. These findings also suggest that phosphorylated HogA plays important roles in septation and conidiation. Accordingly, loss of the SIN component SepH caused reduced expression of HogA-P (Fig 5A and 5F) with completely abolished septation/conidiation, while the deletion of PomA rescued these defects induced by defects of *sepH* as well as suppressing decreased HogA-P expression in the *sepH1 pomA* double mutant (Fig 5F). However, we still could not exclude another possibility for a full-length SepH protein may be also required for the osmotic stimuli-induced septation since *sepH1* is a temperature-sensitive cytokinesis mutant not a null mutant. Our new findings also suggest that the HogA phosphorylation was able to bypass requirement of SepH but not for MobA/SidB since deletion of *pomA* was unable to rescue septation when expression of MobA was turn-off even under the osmotic stress cultural condition. SepH, a key player for early events during cytokinesis while SidB is the last kinase in the core SIN protein kinase cascade. These findings demonstrate that MobA and SidB are required for septation and may also imply HogA-P might be an upstream member of MobA/SidB for septation as shown in the working model of Fig 6.

Taken together, SepH and PomA were able to retroregulate the phosphorylation of HogA for septation and conidiation under non-osmotic stress stimulus conditions. PomA and SepH indirectly affect the expression of HogA. SepH, a main kinase of the SIN, regulates septation/cytokinesis by phosphorylating downstream targets [2]. However, PomA is also a putative kinase and, similar to other serine/threonine protein kinases, the catalytic domain (S-TKc) is included. Thus, we hypothesize that PomA suppresses the defects of SepH indirectly by phosphorylating other proteins. In the absence of PbsB (MAPKK, a putative upstream member of HogA), HogA phosphorylation is completely abolished such that deletion of PomA or SepH is unable to rescue or change the phosphorylation level of HogA. Thus, we conclude that PomA and SepH indirectly regulate HogA phosphorylation through affecting HogA-P upstream kinases.

## Materials and methods

### Strains, media, and culture conditions

All *A*. *nidulans* strains used in this study are summarized in S1 Table. For culture media, in general, *A*. *nidulans* strains were grown on rich media YAG or YUU (YAG supplemented with 5 mM uridine and 10 mM uracil) containing 2% glucose, 0.5% yeast extract, and 1 ml/liter 1,000× trace elements, minimal media PGR or PGRUU (PGR supplemented with 5 mM uridine and 10 mM uracil) or PGRT (PGR supplemented with 100 mM threonine) containing 50 ml/liter salt, 1% glycerol, 0.5 mg/liter pyridoxine, 2.5 mg/liter riboflavin and 1 ml/liter 1,000× trace elements and PDR (PGR replaces the carbon source glycerol with glucose) or PDRUU (PDR supplemented with 5 mM uridine and 10 mM uracil) [40,41]. For strains crossing of *A*. *nidulans*, most of procedure was described as reference [42]. In brief, two parent strains are inoculated for about 5 mm-apart on rich medium YUU for 2 days at 37°C and then digging agars with mixed grown mycelia were transferred to the selective medium incubated for 15 days at room temperature. After that, a matured cleistothecia was picked up and punctured to a 1 ml sterilized H_2_O. Lastly, 10 μl suspension of ascospores was cultured to the rich medium YUU at 37°C and all progenies were scored for phenotypic quantification. Growth conditions, DNA transformation procedures and induction conditions for *alcA(p)*-driven expression were performed as described previously [43,44].

### Construct design and protein tagging

All primers used in this study are shown in S2 Table. To generate the *pomA* deletion cassette, the fusion PCR method was used as previously described. Briefly, approximately 1 kb of the upstream and downstream flanking sequences of the *pomA* gene were amplified using primers pomA-P1/P3 and pomA-P4/P6, respectively, and using the genomic DNA (gDNA) of TN02A7 as a template. As a selectable nutritional marker for fungal transformation, *A*. *fumigatus pyrG* was amplified from the plasmid pXDRFP4 using primers pyrG-F/R. Next, the three aforementioned PCR products were used as templates for amplification using primer pairs pomA-P2/P5 to generate the cassette and was then transformed into the recipient strain TN02A7 [45]. A similar strategy was used to construct the *ankA*, *hogA* and *sidB* deletion mutant. For the generation of *sepH* and *pomA* or *ankA* double mutants, *sepH1* was crossed with *ΔpomA* or *ΔankA*, respectively.

To generate the *pbsB* deletion strain, 5’- and 3’- flanking region fragments of *pbsB* were amplified by using the genomic DNA of TN02A7 as a template and pbsB-p1/p2 and pbsB-p3/p4 as primers, respectively. As a selectable nutritional marker for fungal transformation, the *A*. *nidulans riboB* gene was amplified from the genomic DNA of the parental wild type (R21) using primers Ribo-F/R and then cloned into *p*EASY-Blunt-zero vector (TransGen Biotech, China) to form the plasmid Blunt-ribo. After that, 5’- and 3’- flanking region fragments were ligated into the *Pst1* and *Not1* sites of the plasmid Blunt-ribo, respectively. The resulting plasmid was transformed into the strains of TN02A7, *ΔpomA* and *sepH1*, respectively, to construct the *ΔpbsB*, *ΔpbsB ΔpomA* and *ΔpbsB sepH1* strains and then the expected progenies were obtained by diagnostic PCR.

To generate the RFP-labeled histone H2A strain (ZXA05), *A*. *fumigatus pyrG*, a selectable nutritional marker, and the RFP fragment (except for the termination codon) were amplified from the plasmid pXDRFP4 using primers pyrG-F/R and RFP-F/R, respectively. The H2A DNA fragment (including the termination codon) was amplified using primers H2A-F/R, and the gDNA of TN02A7 was used as a template. Next, the RFP fragment and the H2A DNA fragment were combined by using RFP-F/H2A-R for PCR to form the RFP-H2A fragment. The aforementioned *pyrG* marker and the RFP-H2A fragment were then cloned into the reconstituted vector, pBARGPE1. The *AngpdA* promoter was included sequentially and then the resulting plasmid was transformed into the recipient strain TN02A7 to obtain strain ZXA05. For the generation of RFP-labeled histone H2A in the relative mutant background strains, mutants *sin11*, *sin53* and *ΔpomA* were crossed with the RFP-labeled strain ZXA05, respectively, and then progenies embedding RFP labeling in relative mutants were obtained by diagnostic PCR and microscopic examination.

To generate the *alcA(p)*::GFP-*pomA* strain, an approximate 0.9 kb fragment of the *pomA* gene was amplified from TN02A7 gDNA using primers alc-pomA-F/R. The aforementioned fragment was then cloned into the corresponding sites of pLB01 in which a GFP tag was tagged at the N-terminus of *pomA*. The resulting plasmid was transformed into the recipient strain TN02A7. A similar strategy was used to construct *alc(p)*::GFP-*mobA* strain. For the generation of GFP tag tagged MobA in strains of *sepH1* or *ΔpomA*, *alc(p)*::GFP–*mobA* strain was crossed with *sepH1* or *ΔpomA*, respectively.

To generate constructs for the *pomA* overexpression strain, a *pomA* DNA fragment was amplified by using cla1-pomA-F/R as primers and the gDNA of TN02A7 as a template. The PCR fragment was then cloned into the aforementioned *AngpdA* promoter-included vector pBARGPE1. The resulting plasmid was transformed into the recipient strain TN02A7.

To generate constructs for the site-directed mutant PomA^L1265S^, upstream and downstream flanking sequences were amplified from TN02A7 gDNA by using primers S-pomA-P1/P3 and S-pomA-P4/P6. The upstream flanking sequence with a site directed mutation (Leucine^1265^ was replaced by Serine) was amplified following two steps. First, two fragments were amplified from TN02A7 gDNA with primers S-pomA-P1/S-R and S-F/S-pomA-P3, respectively. Second, the two aforementioned fragments were combined by fusion PCR with primers S-pomA-P1/P3. Finally, a 4.7 kb DNA fragment was generated using primers S-pomA-P2/P5 and the up- and downstream flanking fragments and the *pyrG* marker were used as PCR templates, which were then cloned into the pEasy-Blunt Zero (TransGen Biotech, China) vector. The resulting plasmid was transformed into the recipient strain TN02A7 to obtain mutant PomA^L1265S^. For construction of the double mutant (*sepH* and PomA^L1265S^), the *sepH1* mutant was crossed with PomA^L1265S^ and then the expected progenies were obtained by phenotypic screening and diagnostic PCR.

To generate the HogA phosphorylation sites deletion strains (HogA-ΔP), two DNA fragments were amplified from TN02A7 genomic DNA by using primers HogA-site-p1/HogA-L-de-down and HogA -site-p3/HogA -R-de-up and then these fragments were combined by using HogA-site-p1/HogA-site-p3 for PCR to form the upstream flanking sequence. The downstream flanking sequence was amplified using primers HogA-site-p4/HogA-site-p6 using the genomic DNA (gDNA) of TN02A7 as a template. Lastly, the upstream and downstream flanking sequences and the aforementioned *pyrG* marker were used as templates for amplification using primer pairs HogA-site-p2/HogA-site-p5 to generate the cassette and was then transformed into the recipient strain TN02A7. The strain HogA-ΔP was crossed with *sepH1* to generate constructs of HogA phosphorylation sites deletion in background of *sepH1*.

### Fluorescence microscopy observations

To visualize septa of the indicated strains, conidia were inoculated onto sterile glass coverslips at related temperatures prior to observation. At the detection time point, media on the coverslips were removed and germlings were washed three times with PBS. Cultured cells were then fixed with 4% paraformaldehyde (Polysciences, Warrington, PA, USA) and washed three times with PBS. CFW (Sigma-Aldrich, St. Louis, MO, USA) was used to stain hyphal septa in the dark for 5 min. CFW solution was then removed and the glass coverslip was washed three times with PBS. Images were captured with a Zeiss Axio imager A1 microscope (Zeiss, Jena, Germany) and managed with Adobe Photoshop (Adobe, San Jose, CA, USA).

For visualization of the localization of GFP-PomA and RFP-H2A, a similar strategy procedure was used as described in references.

### RNA isolation and quantitative real-time PCR

To isolate RNA from relative strains, fresh conidia were inoculated on minimal medium in the dark for 24 h at 37 °C and then mycelia were immediately harvested and frozen in liquid nitrogen. Total RNA was extracted using TRIzol (Roche) as described in the manufacturer’s instructions. For gDNA digestion and cDNA synthesis, the HiScriptII Q RT SuperMix for qPCR (+gDNA wiper) Kit (Vazyme) was used following the procedures of the protocol manual [46].

### Protein extraction and Western blot analysis

Fresh conidia were inoculated into minimal medium PGRUU or PDRUU and incubated for 24 h in the dark at related temperatures and then mycelia were harvested and immediately frozen in liquid nitrogen. For protein extraction, the following lysis buffer recipe was used: 10 mM Tris-HCl, pH 7.5, 150 mM NaCl, 0.5 mM EDTA, 0.01% Triton X-100, 1 mM DTT, 1 mM PMSF, and 1:100 protease inhibitor cocktail. A BCA kit was used to quantify the amount of protein according to the manufacturer’s instructions. Lastly, protein samples were loaded onto a 10% SDS polyacrylamide gel and transferred to a PVDF membrane (Immobilon-P, Millipore) in transfer buffer (384 mM glycine, 50 mM Tris, pH 8.4, 20% methanol) at 350 mA for 1.5 h [47]. Next, the membrane was blocked with PBS, 5% milk, and 0.1% Tween 20 and then probed with the following antibodies: anti-phospho-p38 MAPK (The180/Tyr182) antibody (4511T; Cell Signalling Technology; dilution 1:1000) against phosphorylated HogA and anti-p38 (9212S, Cell Signaling Technology; dilution 1:1000) antibody against total MAPK protein and anti-actin antibody (ICN Biomedicals, Inc., clone C4; dilution 1:10000).

### Next-generation sequencing

Conidial spores from *A*. *nidulans* strains S11 and S53 were inoculated into liquid medium and shaken at 42 °C for 18 h at 220 rpm. After harvesting, dried mycelia were then extracted to obtain gDNA and analyzed by NGS, which was performed at Shanghai OE Biotechnology Co., Ltd. as a commercial service. The NGS sequencing raw data submitted to SRA (http://www.ncbi.nlm.nih.gov/sra) at NCBI with accession number SRS4286007.

### Quantitative phosphoproteomics

For quantitative phosphoproteomics, fresh conidia were inoculated into liquid minimal medium PDRUU and incubated for 18 h at 42 °C and shaken at 220 rpm. The harvested mycelia were then dried and extracted to obtain total proteins. The quantitative phosphoproteomics experiment was performed at Hangzhou PTM Biolabs Co., Ltd. as a commercial service. In brief, lysis buffer (8 M urea, 1% Triton-100, 10 mM DTT, and 1% protease inhibitor cocktail) was used and the protein concentration was determined with a BCA kit according to the manufacturer’s instructions. Proteins were digested with trypsin (Promega) and then labeled with a TMT kit (Thermo) according to the manufacturer’s protocol. Next, the labeled tryptic peptides were fractionated by high pH reversed-phase HPLC using a Thermo Betasil C18 column (5 μm particles, 10 mm ID, 250 mm length). After fractioning, the enrichment of phosphorylation was carried out based on biomaterial IMAC microspheres (for proteomics of total proteins, this step was skipped). Next, peptide mixtures were first incubated with an IMAC microsphere suspension and vortexed in loading buffer (50% acetonitrile/6% trifluoroacetic acid) and centrifuged to collect pellets with enriched phosphopeptides in the IMAC microspheres. The IMAC microspheres were then washed with 50% acetonitrile/6% trifluoroacetic acid and 30% acetonitrile/0.1% trifluoroacetic acid to sequentially remove nonspecifically adsorbed peptides. Elution buffer containing 10% NH_4_OH was used to collect the supernatant containing phosphopeptides. For LC-MS/MS analysis, the tryptic peptides were dissolved in 0.1% formic acid (solvent A) and directly loaded onto a homemade reversed-phase analytical column (15-cm length, 75 μm i.d.).

Proteins with more than a 1.2-fold increase or decrease in phosphorylation were analyzed for GO terms and KEGG pathway analysis using the UniProt-GOA database (http://www.ebi.ac.uk/GOA/) and the KEGG database, respectively. The raw phosphoproteomic data have submitted to ProteomeXchange (http://www.ebi.ac.uk/pride/archive/) with accession number PXD012514.

## Supporting information

S1 Fig Colony morphologies of progenies from indicated strains crossed, all strains duplicate cultured on YUU at 30 °C and 42 °C, respectively for 2 days. WT (R21) crossd with (A) S11 and (C) S53, *sepH1* crossed with (B) *sin11* and (D) *sin53*, respectively.
(TIF)

S2 Fig Screening and isolation of the *sepH1* suppressors of septation and conidiation. S11 (A) and S53 (B) were crossed with the wild type strain R21 and then the isolated progenies *sin11* and *sin53* were respectively crossed with *sepH1* in minimal media PDR. All plate colonies used to examine the phenotypes of isolated progenies were cultured in rich media YUU at 30 or 42 °C for 2 days. (C) Hyphal cells stained with CFW for the wild type (R21) and isolated progeny *sepH-1*, *sepH-3*, *sepH sin11-2* and *sepH sin53-2* strains cultured in liquid media at 42 °C for 20 h. Arrows indicate the locations of septa. Bars, 10 μm.
(TIF)

S3 Fig Quantitative data for septation, colony size and conidia production in relative strains. (A) Quantitative data of the conidia production for the WT (TN02A7), *sepH1*, S11 and S53 strains cultured in rich media YUU at 42 °C for 2 days. (B) Quantitative data of septation for the WT (TN02A7), S11, S53, *sepH1 ΔpomA*, *sepH1 pomA*^*L1265S*^ and *sepH1 ΔankA* strains cultured in liquid rich media YUU at 42 °C for 20h. (C) Quantitative data of colony size for the WT (TN02A7), *sepH1*, S11 and S53 strains and (D) WT (R21), *sin11* and *sin53* cultured in rich media YUU at 30 °C and 42 °C for 2 days.
(TIF)

S4 Fig (A) Quantitative data of the colony size for the indicated strains cultured on YAG medium or YAG medium supplemented with 1 M KCl, 1 M NaCl, calcofluor white (CFW) (50 μg/ml), congo red (CR) (100 μg/ml) and caspofungin (1.25 μg/ml) at 37 °C for 2 days. (B) The relative mRNA levels of wild-type (TN02A7) and *OE*::*pomA* strains cultured in minimal medium PDRUU for 24 h.
(TIF)

S5 Fig KEGG pathways enriched in phosphorylated proteins with more than 1.3-fold changes. According to the ratio of fold changes, differentially modified proteins were separated into four parts (name as Q1 to Q4): Q1 (0 < Ratio ≤ 1/1.5), Q2 (1/1.5 < Ratio ≤ 1/1.3), Q3 (1.3 < Ratio ≤ 1.5), Q4 (Ratio > 1.5).
(TIF)

S6 Fig Western blot analysis showing the expression level of HogA-P in the strains of WT, *ΔhogA* and HogA-ΔP cultured in liquid minimal media PGRUU at 37 °C for 24 h. (TIF)

S7 Fig Western blot analysis showing the expression level of HogA-P in the WT, *ΔpbsB*, *ΔpomA ΔpbsB* strains cultured in liquid minimal media PGRUU at 37 °C and *sepH1 ΔpbsB* at 42 °C. (TIF)

S8 Fig MobA and SidB were required for septation under the osmotic-stress condition. (A) (B) (C) Comparison of hyphal cells stained with CFW for the *alc(p)*::GFP-*mobA*, the *ΔsidB*, the *alc(p)*::GFP-*mobA* and *ΔpomA*, *alc(p)*::GFP-*mobA* strains cultured in a de-repressed medium PGR and repressed medium PDR with or without treatment of 1 M NaCl or 1 M KCl at 37 °C for 20 h. Bars, 10 μm. (D) Western blot analysis showing the expression level of HogA-P in strains WT (TN02A7) and *alc(p)*::GFP-*mobA* cultured in minimal medium PDRUU with or without treatment of 1 M NaCl at 37 °C for 20 h. (E) Localization of GFP-MobA in strains ZXA19 and ZXA20 cultured with liquid minimal media PGRT with or without treatment of 1 M NaCl or 1 M KCl at 37 °C for 20 h. The red arrow indicates the septation site and labels for stellate dots indicate the location of SPB. Bars, 10 μm.
(TIF)

S1 Table *A*. *nidulans* strains used in this study. (DOCX)

S2 Table Primers used in this study. (DOCX)

S1 Data File SNP data of S11 and S53. (XLSX)

S2 Data File Quantitative phosphoproteomics data of *sepH1* and *sepH1 ΔpomA*. (XLSX)

## References

[1] Liu B, Morris NR (2000) A spindle pole body-associated protein, SNAD, affects septation and conidiation in *Aspergillus nidulans*. Mol Gen Genet 263: 375–387. 10821171 10.1007/s004380051181

[2] Harris SD (2001) Septum formation in *Aspergillus nidulans*. Curr Opin Microbiol 4: 736–739. 11731327 10.1016/s1369-5274(01)00276-4

[3] Meyer M, Cox JA, Hitchings MDT, Burgin L, Hort MC, et al (2017) Quantifying airborne dispersal routes of pathogens over continents to safeguard global wheat supply. Nat Plants 3: 780–786. 10.1038/s41477-017-0017-5 28947769

[4] Vidal T, Lusley P, Leconte M, de Vallavieille-Pope C, Huber L, et al (2017) Cultivar architecture modulates spore dispersal by rain splash: A new perspective to reduce disease progression in cultivar mixtures. PloS One 12: e0187788 10.1371/journal.pone.0187788 29140990 PMC5687742

[5] Huang MW, Hebert AS, Coon JJ, Hull CM (2015) Protein composition of infectious spores reveals novel sexual development and germination factors in cryptococcus. PloS Genet 11: e1005490 10.1371/journal.pgen.1005490 26313153 PMC4551743

[6] Reiser V, D'Aquino KE, Ee LS, Amon A (2006) The stress-activated mitogen-activated protein kinase signaling cascade promotes exit from mitosis. Mol Biol Cell 17: 3136–3146. 10.1091/mbc.E05-12-1102 16672381 PMC1483046

[7] Yaakov G, Duch A, Garcia-Rubio M, Clotet J, Jimenez J, et al (2009) The stress-activated protein kinase Hog1 mediates S phase delay in response to osmostress. Mol Biol Cell 20: 3572–3582. 10.1091/mbc.E09-02-0129 19477922 PMC2719575

[8] Madrid M, Soto T, Khong HK, Franco A, Vicente J, et al (2006) Stress-induced response, localization, and regulation of the Pmk1 cell integrity pathway in *Schizosaccharomyces pombe*. J Biol Chem 281: 2033–2043. 10.1074/jbc.M506467200 16291757

[9] Bruno KS, Morrell JL, Hamer JE, Staiger CJ (2001) SEPH, a Cdc7p orthologue from *Aspergillus nidulans*, functions upstream of actin ring formation during cytokinesis. Mol Microbiol 42: 3–12. 11679062 10.1046/j.1365-2958.2001.02605.x

[10] Krapp A, Simanis V (2008) An overview of the fission yeast septation initiation network (SIN). Biochem Soc Trans 36: 411–415. 10.1042/BST0360411 18481970

[11] Yu ZZ, Armant O, Fischer R (2016) Fungi use the SakA (HogA) pathway for phytochrome-dependent light signalling. Nat Microbiol 1: 16019 10.1038/nmicrobiol.2016.19 27572639

[12] Ma D, Li R (2013) Current understanding of HOG-MAPK pathway in *Aspergillus fumigatus*. Mycopathologia 175: 13–23. 10.1007/s11046-012-9600-5 23161019

[13] Yaffe MB, Elia AE (2001) Phosphoserine/threonine-binding domains. Curr Opin Cell Biol 13: 131–138. 11248545 10.1016/s0955-0674(00)00189-7

[14] Lara-Rojas F, Sanchez O, Kawasaki L, Aguirre J (2011) *Aspergillus nidulans* transcription factor AtfA interacts with the MAPK SakA to regulate general stress responses, development and spore functions. Mol Microbiol 80: 436–454. 10.1111/j.1365-2958.2011.07581.x 21320182 PMC3108070

[15] Soto T, Villar-Tajadura MA, Madrid M, Vicente J, Gacto M, et al (2010) Rga4 modulates the activity of the fission yeast cell integrity MAPK pathway by acting as a Rho2 GTPase-activating protein. J Biol Chem 285: 11516–11525. 10.1074/jbc.M109.071027 20164182 PMC2857030

[16] Serrano R, Martin H, Casamayor A, Arino J (2006) Signaling alkaline pH stress in the yeast *Saccharomyces cerevisiae* through the Wsc1 cell surface sensor and the Slt2 MAPK pathway. J Biol Chem 281: 39785–39795. 10.1074/jbc.M604497200 17088254

[17] Martinez-Salgado JL, Leon-Ramirez CG, Pacheco AB, Ruiz-Herrera J, de la Rosa AP (2013) Analysis of the regulation of the Ustilago maydis proteome by dimorphism, pH or MAPK and GCN5 genes. J Proteomics 79: 251–262. 10.1016/j.jprot.2012.12.022 23305952

[18] Cuenda A, Rousseau S (2007) p38 MAP-kinases pathway regulation, function and role in human diseases. Biochim Biophys Acta 1773: 1358–1375. 10.1016/j.bbamcr.2007.03.010 17481747

[19] Pigula A, Drubin DG, Barnes G (2014) Regulation of mitotic spindle disassembly by an environmental stress-sensing pathway in budding yeast. Genetics 198: 1043–1057. 10.1534/genetics.114.163238 25213170 PMC4224151

[20] Kawasaki L, Sanchez O, Shiozaki K, Aguirre J (2002) SakA MAP kinase is involved in stress signal transduction, sexual development and spore viability in *Aspergillus nidulans*. Mol Microbiol 45: 1153–1163. 12180932 10.1046/j.1365-2958.2002.03087.x

[21] de Castro PA, dos Reis TF, Dolan SK, Manfiolli AO, Brown NA, et al (2016) The *Aspergillus fumigatus* SchA(SCH9) kinase modulates SakA(HOG1) MAP kinase activity and it is essential for virulence. Mol Microbiol 102: 642–671. 10.1111/mmi.13484 27538790 PMC5207228

[22] Zhong G, Wei W, Guan Q, Ma Z, Wei H, et al (2012) Phosphoribosyl pyrophosphate synthetase, as a suppressor of the *sepH* mutation in *Aspergillus nidulans*, is required for the proper timing of septation. Mol Microbiol 86: 894–907. 10.1111/mmi.12026 22994198

[23] Savitski MM, Mathieson T, Zinn N, Sweetman G, Doce C, et al (2013) Measuring and managing ratio compression for accurate iTRAQ/TMT quantification. J Proteome Res 12: 3586–3598. 10.1021/pr400098r 23768245

[24] Grallert A, Connolly Y, Smith DL, Simanis V, Hagan IM (2012) The *S-pombe* cytokinesis NDR kinase Sid2 activates Fin1 NIMA kinase to control mitotic commitment through Pom1/Wee1. Nat Cell Biol 14: 738–745. 10.1038/ncb2514 22684255 PMC4284365

[25] Allard CAH, Opalko HE, Liu KW, Medoh U, Moseley JB (2018) Cell size-dependent regulation of Wee1 localization by Cdr2 cortical nodes. J Cell Biol 217: 1589–1599. 10.1083/jcb.201709171 29514920 PMC5940308

[26] Deng L, Baldissard S, Kettenbach AN, Gerber SA, Moseley JB (2014) Dueling kinases regulate cell size at division through the SAD kinase Cdr2. Curr Biol 24: 428–433. 10.1016/j.cub.2014.01.009 24508166 PMC4055034

[27] Guzman-Vendrell M, Rincon SA, Dingli F, Loew D, Paoletti A (2015) Molecular control of the Wee1 regulatory pathway by the SAD kinase Cdr2. J Cell Sci 128: 2842–2853. 10.1242/jcs.173146 26071525

[28] Ye XS, Fincher RR, Tang A, Osmani SA (1997) The G2/M DNA damage checkpoint inhibits mitosis through Tyr15 phosphorylation of p34cdc2 in *Aspergillus nidulans*. EMBO J 16: 182–192. 10.1093/emboj/16.1.182 9009279 PMC1169625

[29] Kraus PR, Harris SD (2001) The *Aspergillus nidulans* snt genes are required for the regulation of septum formation and cell cycle checkpoints. Genetics 159: 557 11606533 10.1093/genetics/159.2.557 PMC1461812

[30] Sharpless KE, Harris SD (2002) Functional characterization and localization of the *Aspergillus nidulans* formin SEPA. Mol Biol Cell 13: 469–479. 10.1091/mbc.01-07-0356 11854405 PMC65642

[31] Kim JM, Lu L, Shao R, Chin J, Liu B (2006) Isolation of mutations that bypass the requirement of the septation initiation network for septum formation and conidiation in *Aspergillus nidulans*. Genetics 173: 685–696. 10.1534/genetics.105.054304 16624915 PMC1526526

[32] Martin SG, Berthelot-Grosjean M (2009) Polar gradients of the DYRK-family kinase Pom1 couple cell length with the cell cycle. Nature 459: 852–856. 10.1038/nature08054 19474792

[33] Bhatia P, Hachet O, Hersch M, Rincon SA, Berthelot-Grosjean M, et al (2014) Distinct levels in Pom1 gradients limit Cdr2 activity and localization to time and position division. Cell Cycle 13: 538–552. 10.4161/cc.27411 24316795

[34] Kettenbach AN, Deng L, Wu YJ, Baldissard S, Adamo ME, et al (2015) Quantitative phosphoproteomics reveals pathways for coordination of cell growth and division by the conserved fission yeast kinase Pom1. MolCell Proteomics 14: 1275–1287. 10.1074/mcp.M114.045245 PMC4424399 25720772

[35] Padte NN, Martin SG, Howard M, Chang F (2006) The cell-end factor Pom1p inhibits Mid1p in specification of the cell division plane in fission yeast. Curr Biol 16: 2480–2487. 10.1016/j.cub.2006.11.024 17140794

[36] Bahler J, Pringle JR (1998) Pom1p, a fission yeast protein kinase that provides positional information for both polarized growth and cytokinesis. Genes Dev 12: 1356–1370. 10.1101/gad.12.9.1356 9573052 PMC316787

[37] Bahler J, Nurse P (2001) Fission yeast Pom1p kinase activity is cell cycle regulated and essential for cellular symmetry during growth and division. EMBO J 20: 1064–1073. 10.1093/emboj/20.5.1064 11230130 PMC145493

[38] Hachet O, Berthelot-Grosjean M, Kokkoris K, Vincenzetti V, Moosbrugger J, et al (2011) A phosphorylation cycle shapes gradients of the DYRK family kinase Pom1 at the plasma membrane. Cell 145: 1116–1128. 10.1016/j.cell.2011.05.014 21703453

[39] Niccoli T, Arellano M, Nurse P (2003) Role of Tea1p, Tea3p and Pom1p in the determination of cell ends in *Schizosaccharomyces pombe*. Yeast 20: 1349–1358. 10.1002/yea.1054 14663827

[40] Gupta SK, Maggon KK, Venkitasubramanian TA (1976) Effect of zinc on adenine nucleotide pools in relation to aflatoxin biosynthesis in *Aspergillus parasiticus*. Appl Environ Microbiol 32: 753–756. 1008554 10.1128/aem.32.6.753-756.1976 PMC170456

[41] Jiang P, Wei WF, Zhong GW, Zhou XG, Qiao WR, et al (2017) The function of the three phosphoribosyl pyrophosphate synthetase (Prs) genes in hyphal growth and conidiation in *Aspergillus nidulans*. Microbiology 163: 218–232. 10.1099/mic.0.000427 28277197

[42] Todd RB, Davis MA, Hynes MJ (2007) Genetic manipulation of *Aspergillus nidulans*: heterokaryons and diploids for dominance, complementation and haploidization analyses. Nat Protoc 2: 822–830. 10.1038/nprot.2007.113 17446882

[43] Liu B, Xiang X, Lee YRJ (2003) The requirement of the LC8 dynein light chain for nuclear migration and septum positioning is temperature dependent in *Aspergillus nidulans*. Mol Microbiol 47: 291–301. 12519184 10.1046/j.1365-2958.2003.03285.x

[44] Osmani SA, Pu RT, Morris NR (1988) Mitotic induction and maintenance by overexpression of a G2-specific gene that encodes a potential protein kinase. Cell 53: 237–244. 3359487 10.1016/0092-8674(88)90385-6

[45] Yu JH, Hamari Z, Han KH, Seo JA, Reyes-Dominguez Y, et al (2004) Double-joint PCR: a PCR-based molecular tool for gene manipulations in filamentous fungi. Fungal Genet Biol 41: 973–981. 10.1016/j.fgb.2004.08.001 15465386

[46] Long N, Orasch T, Zhang S, Gao L, Xu X, et al (2018) The Zn2Cys6-type transcription factor LeuB cross-links regulation of leucine biosynthesis and iron acquisition in *Aspergillus fumigatus*. PLoS Genet 14: e1007762 10.1371/journal.pgen.1007762 30365497 PMC6221358

[47] Zhang Y, Zheng Q, Sun C, Song J, Gao L, et al (2016) Palmitoylation of the cysteine residue in the DHHC motif of a palmitoyl transferase mediates Ca2+ homeostasis in *Aspergillus*. PLoS Genet 12: e1005977 10.1371/journal.pgen.1005977 27058039 PMC4825924

