# Supplementary figures and images for: A new identified suppressor of Cdc7p/SepH kinase, PomA, regulates fungal asexual reproduction via affecting phosphorylation of MAPK-HogA
Source: PLoS Genet. 2019 Jun 13;15(6):e1008206. doi: 10.1371/journal.pgen.1008206 (PMC6592577; doi:10.1371/journal.pgen.1008206)

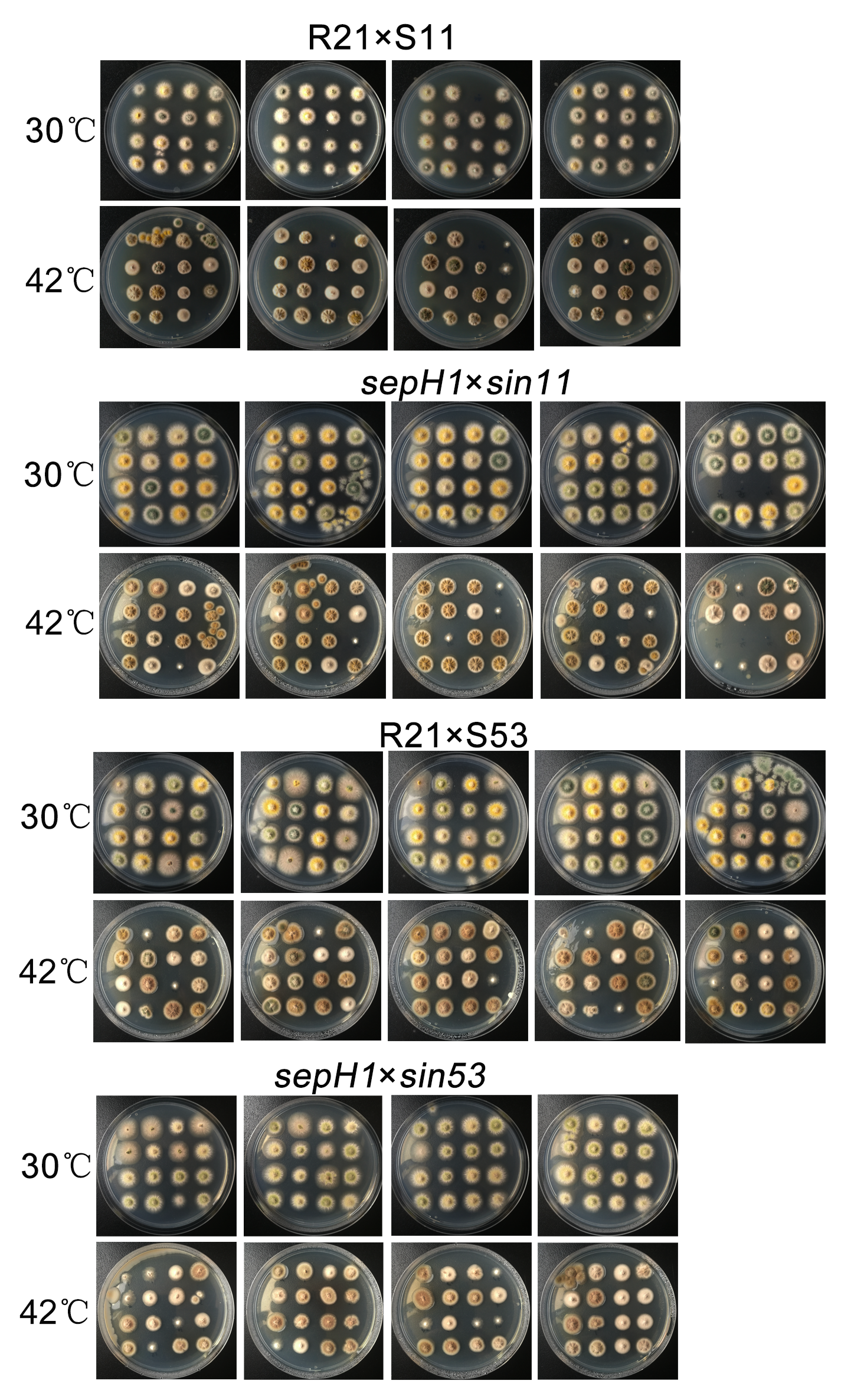

Supplement: S1 Fig — WT (R21) crossd with (A) S11 and (C) S53, sepH1 crossed with (B) sin11 and (D) sin53, respectively. (TIF) [file pgen.1008206.s001.tif]

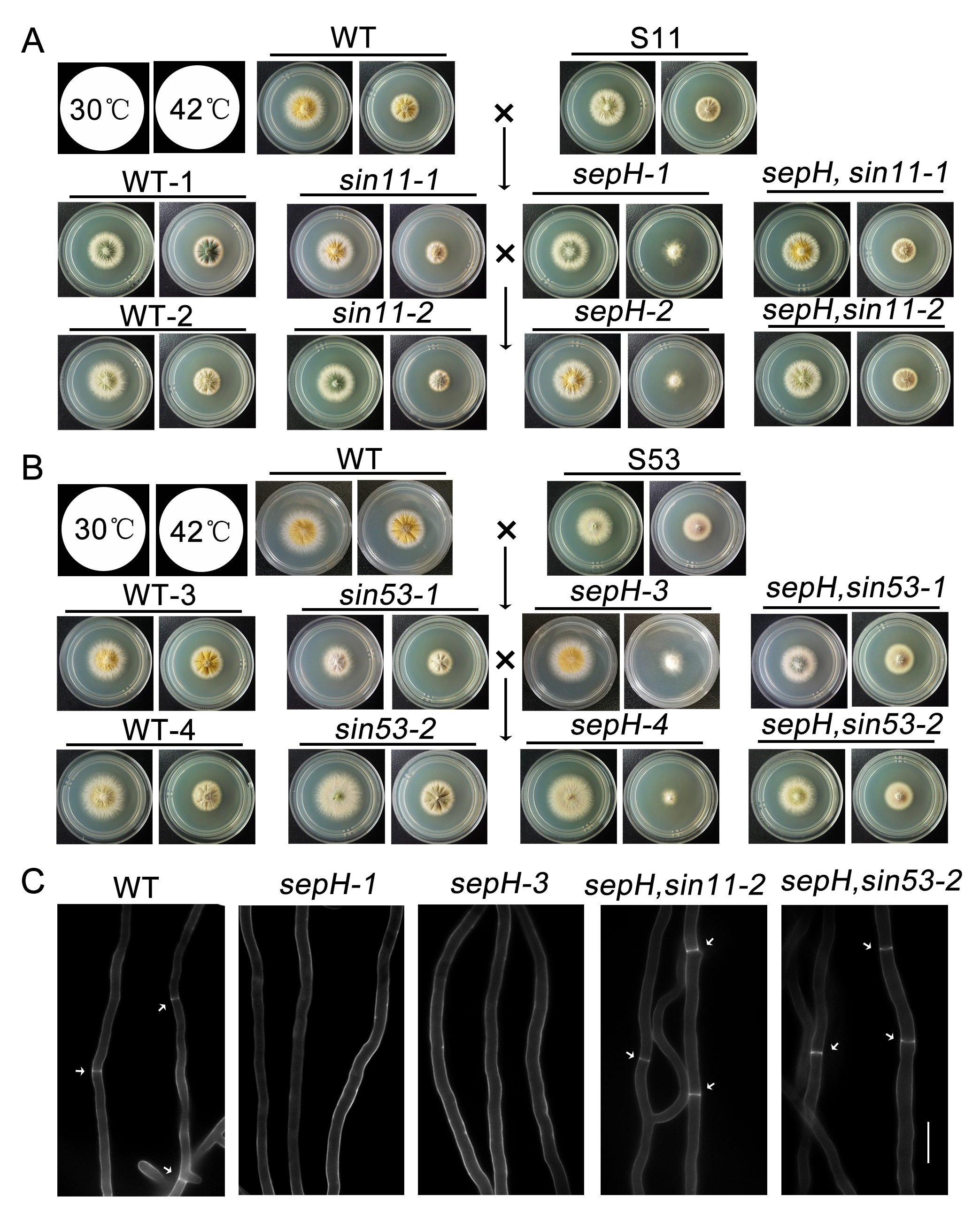

Supplement: S2 Fig — S11 (A) and S53 (B) were crossed with the wild type strain R21 and then the isolated progenies sin11 and sin53 were respectively crossed with sepH1 in minimal media PDR. All plate colonies used to examine the phenotypes of isolated progenies were cultured in rich media YUU at 30 or 42 °C for 2 days. (C) Hyphal cells stained with CFW for the wild type (R21) and isolated progeny sepH-1, sepH-3, sepH sin11-2 and sepH sin53-2 strains cultured in liquid media at 42 °C for 20 h. Arrows indicate the locations of septa. Bars, 10 μm. (TIF) [file pgen.1008206.s002.tif]

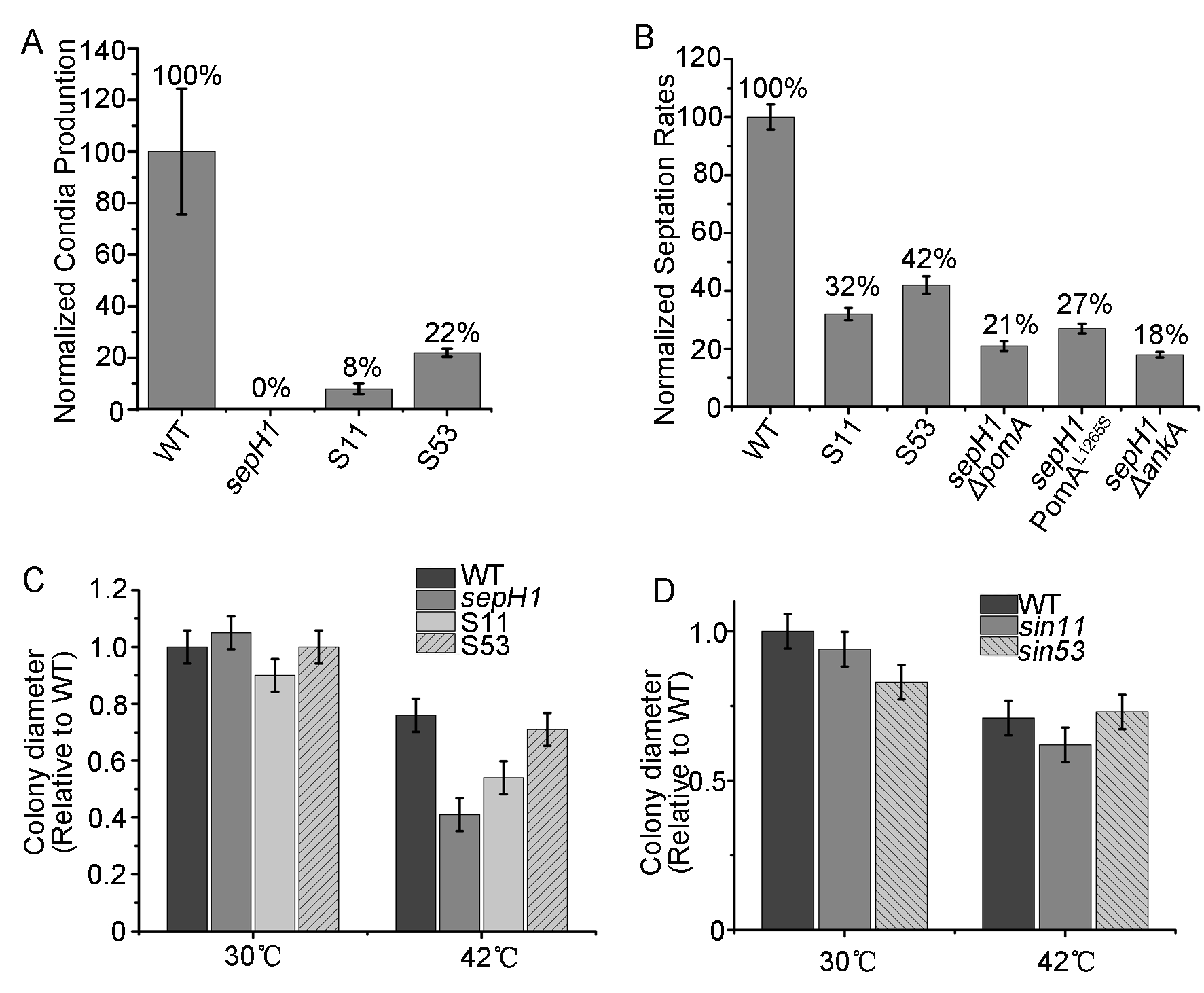

Supplement: S3 Fig — (A) Quantitative data of the conidia production for the WT (TN02A7), sepH1, S11 and S53 strains cultured in rich media YUU at 42 °C for 2 days. (B) Quantitative data of septation for the WT (TN02A7), S11, S53, sepH1 ΔpomA, sepH1 pomAL1265S and sepH1 ΔankA strains cultured in liquid rich media YUU at 42 °C for 20h. (C) Quantitative data of colony size for the WT (TN02A7), sepH1, S11 and S53 strains and (D) WT (R21), sin11 and sin53 cultured in rich media YUU at 30 °C and 42 °C for 2 days. (TIF) [file pgen.1008206.s003.tif]

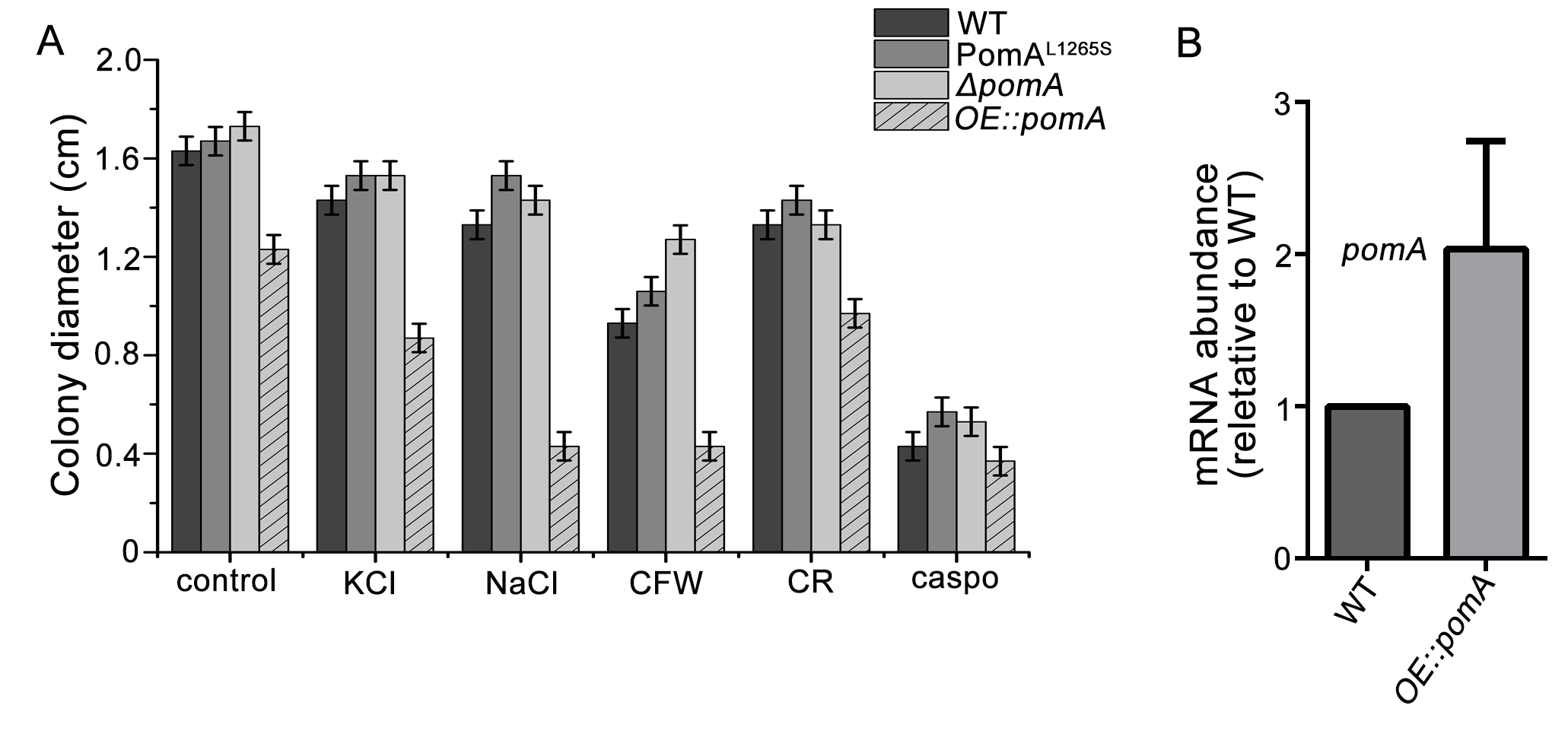

Supplement: S4 Fig — (A) Quantitative data of the colony size for the indicated strains cultured on YAG medium or YAG medium supplemented with 1 M KCl, 1 M NaCl, calcofluor white (CFW) (50 μg/ml), congo red (CR) (100 μg/ml) and caspofungin (1.25 μg/ml) at 37 °C for 2 days. (B) The relative mRNA levels of wild-type (TN02A7) and OE::pomA strains cultured in minimal medium PDRUU for 24 h. (TIF) [file pgen.1008206.s004.tif]

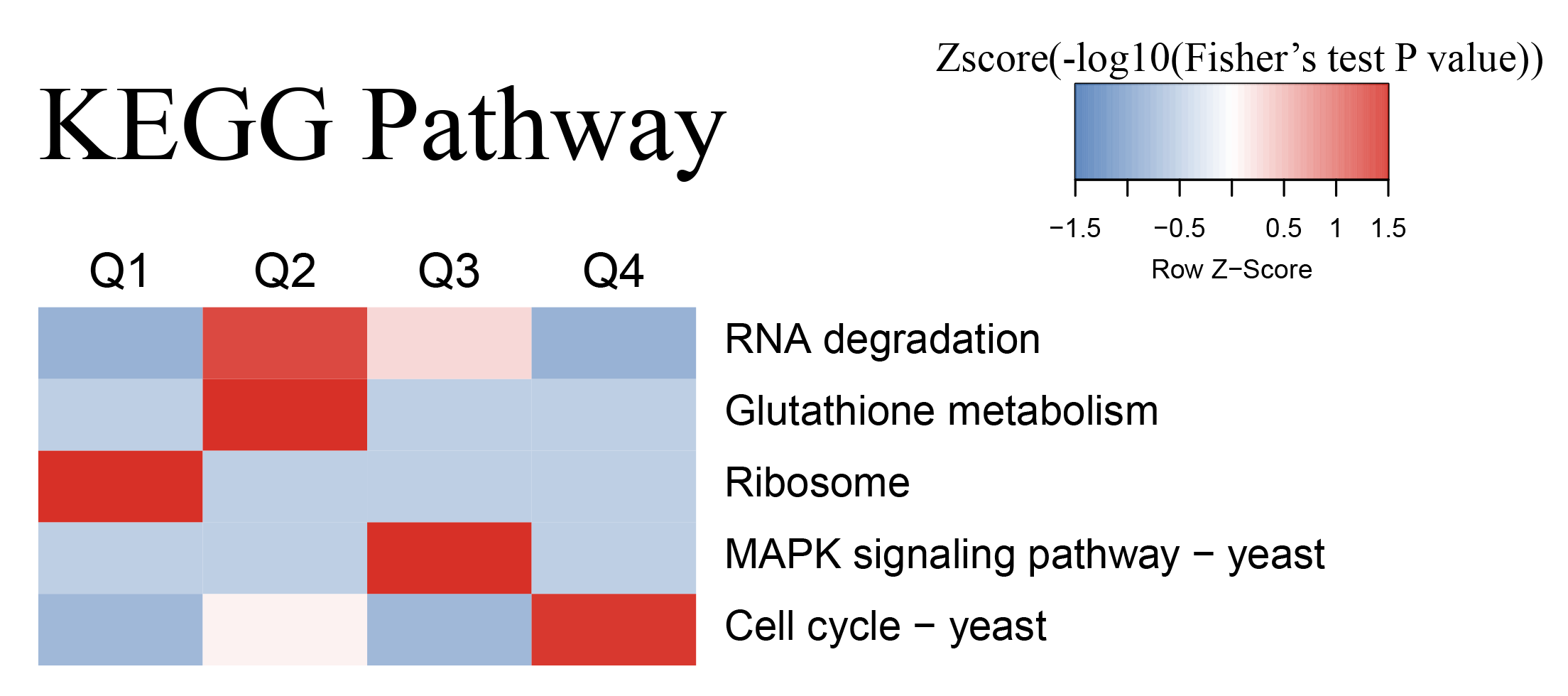

Supplement: S5 Fig — According to the ratio of fold changes, differentially modified proteins were separated into four parts (name as Q1 to Q4): Q1 (0 < Ratio ≤ 1/1.5), Q2 (1/1.5 < Ratio ≤ 1/1.3), Q3 (1.3 < Ratio ≤ 1.5), Q4 (Ratio > 1.5). (TIF) [file pgen.1008206.s005.tif]

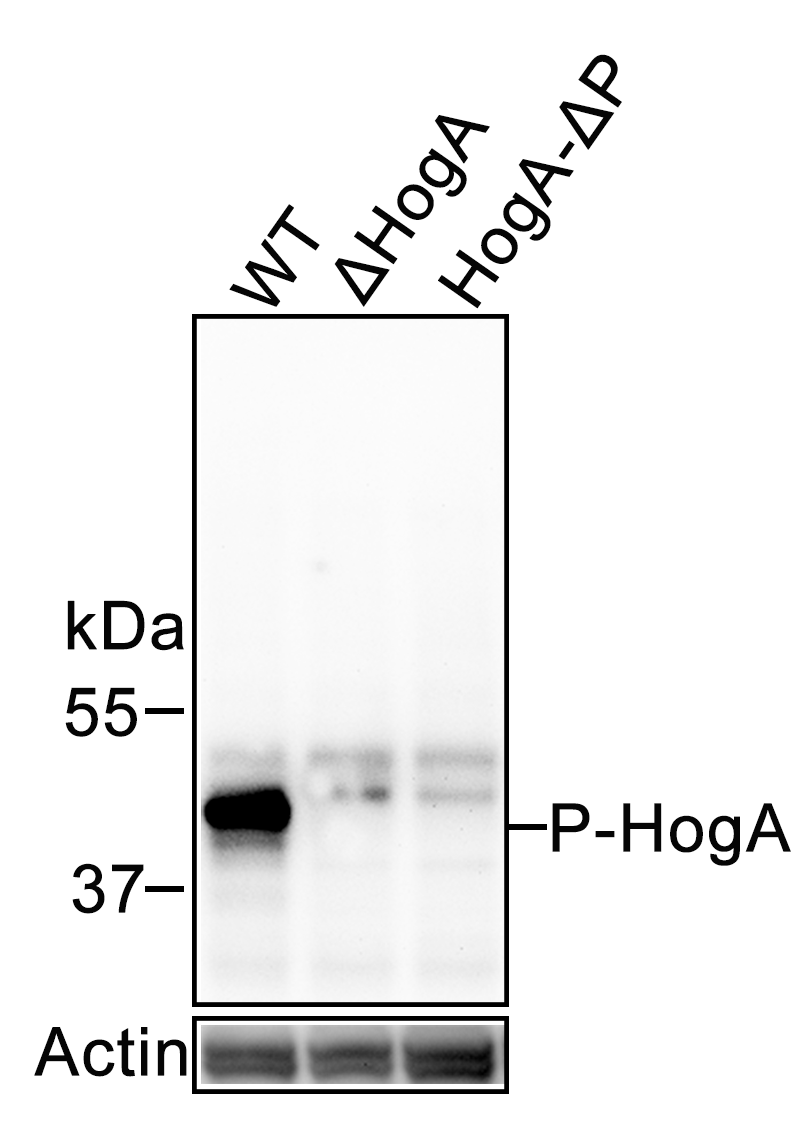

Supplement: S6 Fig — (TIF) [file pgen.1008206.s006.tif]

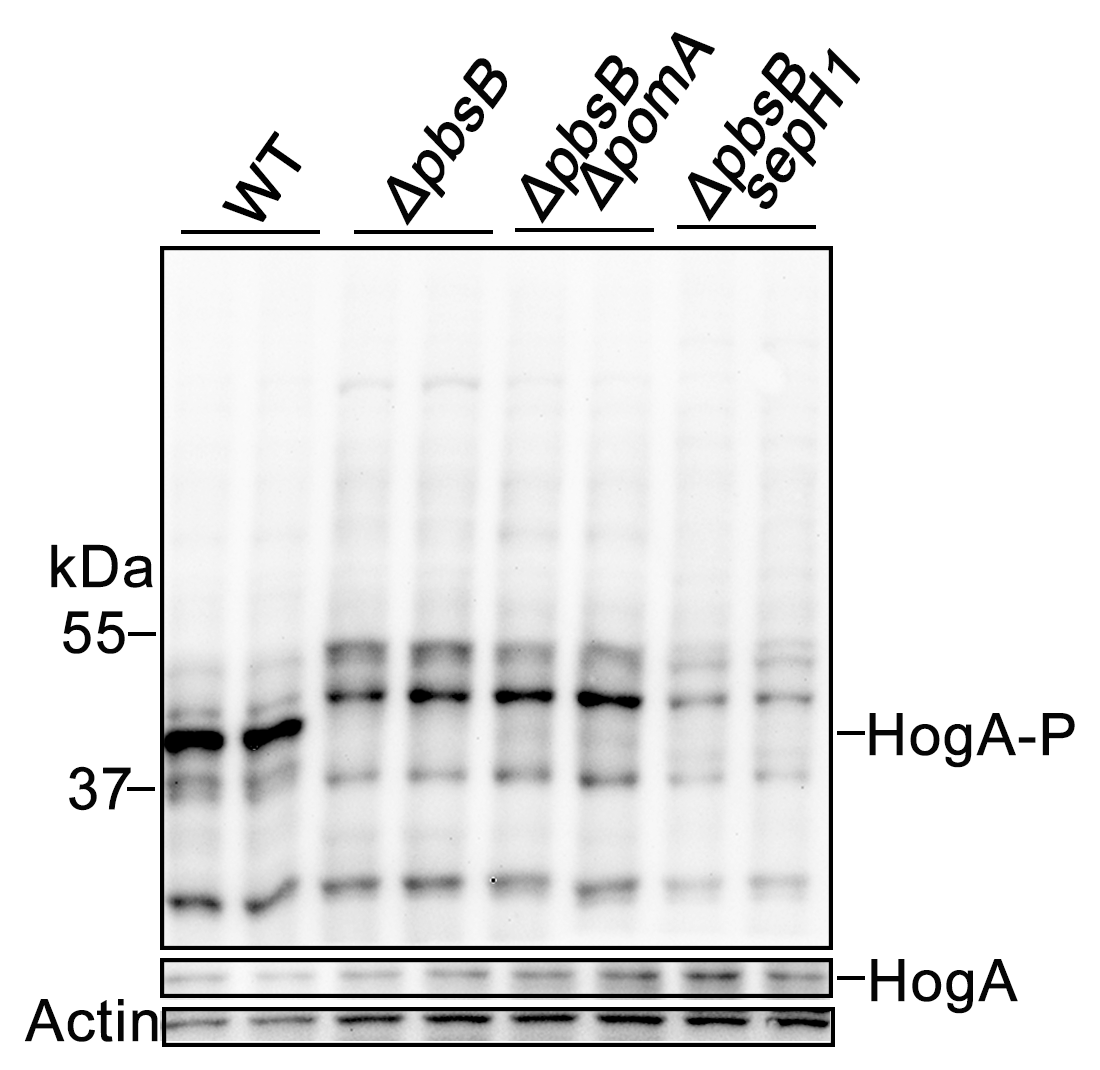

Supplement: S7 Fig — (TIF) [file pgen.1008206.s007.tif]

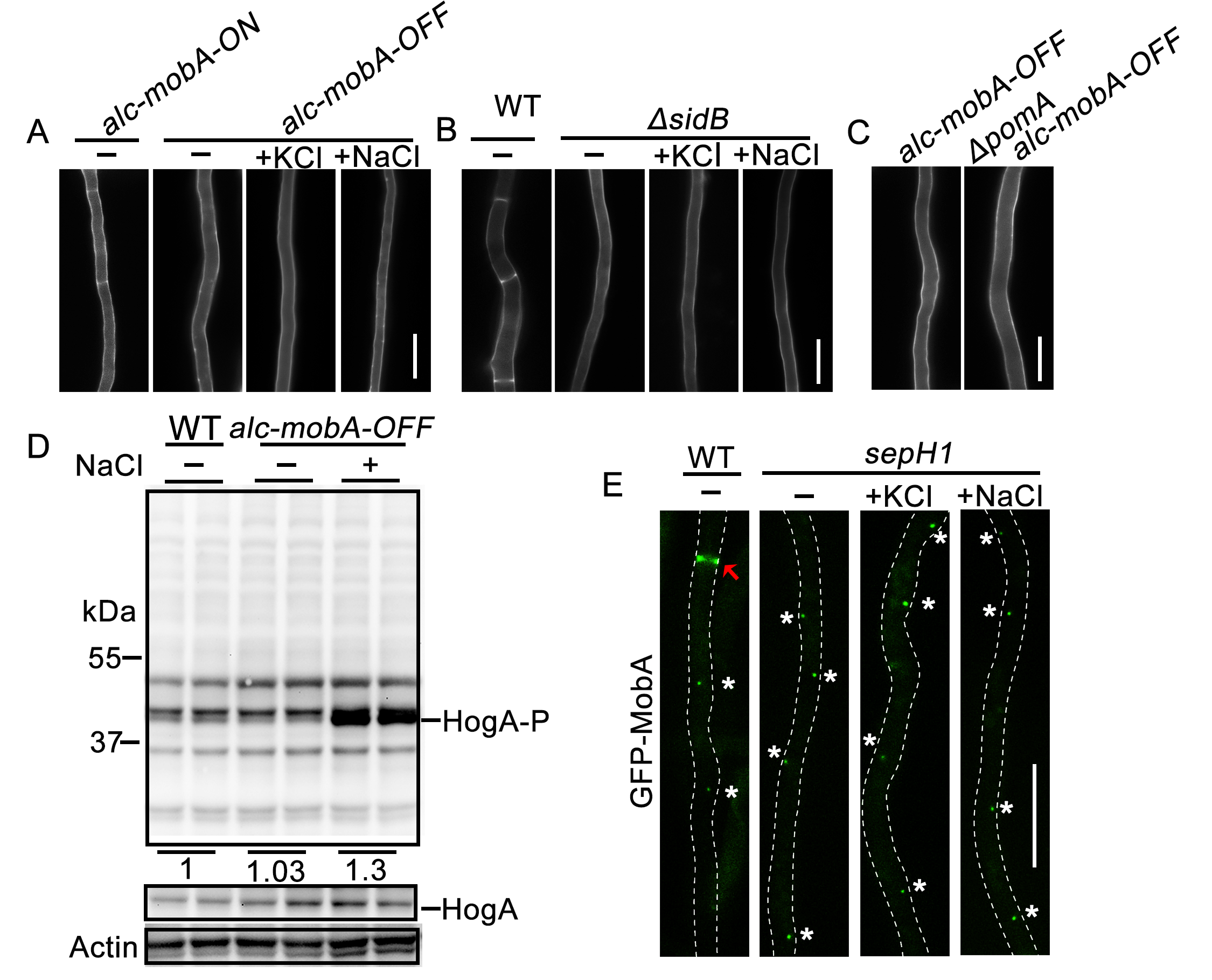

Supplement: S8 Fig — (A) (B) (C) Comparison of hyphal cells stained with CFW for the alc(p)::GFP-mobA, the ΔsidB, the alc(p)::GFP-mobA and ΔpomA, alc(p)::GFP-mobA strains cultured in a de-repressed medium PGR and repressed medium PDR with or without treatment of 1 M NaCl or 1 M KCl at 37 °C for 20 h. Bars, 10 μm. (D) Western blot analysis showing the expression level of HogA-P in strains WT (TN02A7) and alc(p)::GFP-mobA cultured in minimal medium PDRUU with or without treatment of 1 M NaCl at 37 °C for 20 h. (E) Localization of GFP-MobA in strains ZXA19 and ZXA20 cultured with liquid minimal media PGRT with or without treatment of 1 M NaCl or 1 M KCl at 37 °C for 20 h. The red arrow indicates the septation site and labels for stellate dots indicate the location of SPB. Bars, 10 μm. (TIF) [file pgen.1008206.s008.tif]
