# Supplementary material for: A new identified suppressor of Cdc7p/SepH kinase, PomA, regulates fungal asexual reproduction via affecting phosphorylation of MAPK-HogA
Source: PLoS Genet. 2019 Jun 13;15(6):e1008206. doi: 10.1371/journal.pgen.1008206 (PMC6592577; doi:10.1371/journal.pgen.1008206)
Supplement: S1 Table — (DOCX) [file pgen.1008206.s009.docx]

| **Strain** | ***Genotype*** | **source** |
| --- | --- | --- |
| R21 | *pabaA1;yA2* | FGSC |
| WJA01 | *pyroA4,nkuA::argB2;riboB2;veA1* | ([*42*](#_ENREF_42)) |
| TN02A7 | *pyrG89; pyroA4;nkuA::argB2;riboB2;veA1* | ([*43*](#_ENREF_43)) |
| *sepH1* | *pyrG89; veA1;sepH1;chaA1* | ([*18*](#_ENREF_18)) |
| ZXA01 | *pyrG89; veA1;sepH1;chaA1* | this study |
| ZXA02 | *pyrG89; veA1;sepH1;chaA1* | this study |
| ZXA03 | *pabaA1;yA2* | this study |
| ZXA04 | *pabaA1* | this study |
| ZXA05 | *pyroA4;nkuA::argB2; RFP-H2A::pyrG89;riboB2;veA1* | this study |
| ZXA06 | *RFP-H2A:: pabaA1;yA2* | this study |
| ZXA07 | *RFP-H2A:: pabaA1* | this study |
| ZXA08 | *ΔpomA::pyrG89; pyroA4;nkuA::argB2;riboB2;veA1* | this study |
| ZXA09 | *pomA^L1265S^::pyrG89; pyroA4;nkuA::argB2;riboB2;veA1* | this study |
| ZXA10 | *ΔpomA::pyrG89; pyroA4;nkuA::argB2;riboB2;sepH1;veA1* | this study |
| ZXA11 | *pomA^L1265S^::pyrG89; pyroA4;nkuA::argB2;riboB2;sepH1;veA1* | this study |
| ZXA12 | *pyrG89; gpd(p)-pomA-pyroA4; pyroA4;nkuA::argB2;riboB2;veA1* | this study |
| ZXA13 | *ΔpomA::pyrG89; RFP-H2A;pyroA4;nkuA::argB2;riboB2;veA1* | this study |
| ZXA14 | *pyrG89; alcA(p)-pomA -pyr4;pyroA4;nkuA::argB2; riboB2;veA1* | this study |
| ZXA15 | *ΔhogA:: pyrG89; pyroA4;nkuA::argB2;riboB2;veA1* | this study |
| ZXA16 | *ΔankA:: pyrG89; pyroA4;nkuA::argB2;riboB2;veA1* | this study |
| ZXA17 | *ΔankA:: pyrG89; pyroA4;sepH1;nkuA::argB2;riboB2;veA1* | this study |
| ZXA18 | *ΔsidB::pyrG89;. pyroA4;nkuA::argB2;riboB2;veA1* | this study |
| ZXA19 | *alcA(p)::GFP-MobA::pyr4;pyrG89; pyroA4;nkuA::argB2;riboB2;veA1* | this study |
| ZXA20 | *alcA(p)::GFP-MobA::pyr4;pyrG89;pyroA4;nkuA::argB2;riboB2; sepH1;veA1* | this study |
| ZXA21 | *alcA(p)::GFP-MobA::pyr4;ΔpomA::pyrG89;pyroA4;nkuA::argB2;riboB2;veA1* | this study |
| ZXA22 | *hogA^Δ171-173^::pyrG89; pyroA4;nkuA::argB2;riboB2;veA1* | this study |
| ZXA23 | *hogA^T171D Y173D^::pyrG89; pyroA4;nkuA::argB2;riboB2;veA1* | this study |
| ZXA24 | *ΔhogA:: pyrG89; pyroA4;nkuA::argB2;riboB2; sepH1;veA1* | this study |
| ZXA25 | *hogA^Δ171-173^::pyrG89; pyroA4;nkuA::argB2;riboB2; sepH1;veA1* | this study |
| ZXA26 | *hogA^T171D Y173D^::pyrG89; pyroA4;nkuA::argB2;riboB2; sepH1;veA1* | this study |
| ZXA27 | *ΔpbsB:: riboB2;pyrG89; pyroA4;nkuA::argB2;veA1* | this study |
| ZXA28 | *ΔpbsB:: riboB2;ΔpomA::pyrG89; pyroA4;nkuA::argB2; veA1* | this study |
| ZXA29 | *ΔpbsB:: riboB2; pyrG89; veA1;sepH1;chaA1* | this study |
| ZXA30 | *veA1* | this study |
| ZXA31 | *pyrG89; veA1; chaA1* | this study |
| ZXA32 | *pabaA1;yA2* | this study |
| ZXA33 | *pyrG89; pabaA1;yA2* | this study |
| ZXA34 | *pomA^L1265S^; veA1* | this study |
| ZXA35 | *veA1;chaA1* | this study |
| ZXA36 | *pyroA4;riboB2;veA1;sepH1* | this study |
| ZXA37 | *pyroA4;riboB2;veA1;sepH1; yA2* | this study |
| ZXA38 | *pyrG89; pabaA1; sepH1;yA2* | this study |
| ZXA39 | *pyrG89;veA1;sepH1* | this study |
| ZXA40 | *pabaA1;sepH1;pyroA4;riboB2; yA2* | this study |
| ZXA41 | *sepH1 ;veA1; chaA1* | this study |
| ZXA42 | *sepH1;pomA;pyroA4;riboB2;* | this study |
| ZXA43 | *pyrG89; sepH1;pomA* | this study |
