# Supplementary material for: A new identified suppressor of Cdc7p/SepH kinase, PomA, regulates fungal asexual reproduction via affecting phosphorylation of MAPK-HogA
Source: PLoS Genet. 2019 Jun 13;15(6):e1008206. doi: 10.1371/journal.pgen.1008206 (PMC6592577; doi:10.1371/journal.pgen.1008206)
Supplement: S2 Table — (DOCX) [file pgen.1008206.s010.docx]

| Primer name | DNA sequence 5′-3′ |
| --- | --- |
| RFP-F | CCTTTAATCAAGCTTATCGATATGGCCTCCTCCGAGGACGTC |
| RFP-R | GCCACCAGATTTGCCGCCAGTGGCGCCGGTGGAGTGGCGGCCC |
| H2A-F | ACTGGCGGCAAATCTGGTGGC |
| H2A-R | CTCGAGGTCGACGGTATCGATTTACAGCTCCTGGCTGCCCTTTCC |
| pyrG-F | GCCTCAAACAATGCTCTTCACC |
| pyrG-R | CTGTCTGAGAGGAGGCCTGATG |
| pabaA-F | GGCTGCAGGTCGAGCGGCCGCACTGCCTCGTTACCTGATCC |
| pabaA-R | GCATAGTACCGAGAAACTAGTCGAGCTTCATAAAGCCTTGG |
| pomA-p1 | GTAGTACGACCAGTAGATGG |
| pomA-p2 | TCAGAGGAAGACTGAAAGCCG |
| pomA-p3 | CTCTAGATGCATGCTCGAGCGTCACACTTCGGTCGGGGAA |
| pomA-p4 | CAGTGCCTCCTCTCAGACAGGAACATTGCATGTCTGCCATG |
| pomA-p5 | AACCGTGGACCAGAAAAAGG |
| pomA-p6 | GCACGCCATCAATTAGCATC |
| pomA-F | CTCAAGTCTAGGCGGCAATC |
| pomA-R | CCATTCTGTGACCCGTCATG |
| S- pomA-p1 | CCGATCCTGATAGCAATGG |
| S- pomA-p2 | CCTAAGGATCATGTCAATCC |
| S- pomA-p3 | GGTGAAGAGCATTGTTTGAGGCTCAAGTGAGGGTATAAGAAG |
| S- pomA-p4 | CATCAGTGCCTCCTCTCAGACAGCATTGCATGTCTGCCATG |
| S- pomA-p5 | AGCTTGTCGGCTCCTTCTTC |
| S- pomA-p6 | CCATAAATCCGACATGAGTC |
| S-F | GCAGGTTTCGAAGTGTGAC |
| S-R | GTCACACTTCGAAACCTGC |
| alc-pomA-F | ATATGGCGCCGCTGCCGGAAAAGCTGTCGGAC |
| alc-pomA-R | GGACTAGTGGCTCTGGCGACGATTTCG |
| cla-pomA-F | CCTTTAATCAAGCTTATCGATATGCCGGAAAAGCTGTCGG |
| cla-pomA-R | CTCGAGGTCGACGGTATCGATTCATTTGGGCTTCTATCTTG |
| RT-pomA-F | GACATGTGGAGTTTGGGATGC |
| RT-pomA-R | GCTCTTTTGAGCTAGGGCGT |
| Rt-tubulin-F | GCCGGTATGGGTACTCTTTTG |
| Rt-tubulin-R | GTCTCATCGGAGTGCTCAACG |
| ankA-p1 | AGAATCTGGGCAGTGGGAAC |
| ankA-p2 | CACATAGTCACGGTCCATGAC |
| ankA-p3 | CTATTATCTGACTTACCCGCCAACGTTGACTAGATCCACGCA |
| ankA-p4 | CCAAGAGAAAGCGTCAAGTCAG CAGTACAATCTACGACGG |
| ankA-p5 | GGTCGAATTATCTCGAGGGC |
| ankA-p6 | CTATCCTCTAGCCGTTCAAG |
| ankA-F | CTAACACACCCCAACCCCAT |
| ankA-R | AAACTCTCCAGTGCCCACAAG |
| HogA-p1 | GAGGATGAGTACATCGACGAC |
| HogA-p2 | GTCCACAGACCAAAGATCAG |
| HogA-p3 | GGTGAAGAGCATTGTTTGAGGCCCTGCTTGCAAGATGAGTGAG |
| HogA -p4 | CATCAGTGCCTCCTCTCAGACAGAGCCCTAATGAATCGCGTGG |
| HogA -p5 | GGATTTTCCGTCACCACAAC |
| HogA -p6 | CTAATCACCAAACCTTCCCG |
| HogA -F | AATGGGTGCTTTTGGACTGG |
| HogA -R | ATCGTGGTATGGCGCTAGGT |
| HogA -site-p1 | ATGGCGGAATTTGTACGTGC |
| HogA -site-p2 | CGTCCTGGAATGCATTCTGAC |
| HogA -site-p3 | GGTGAAGAGCATTGTTTGAGGCGGCTTTATTGGAAACCTTGCTGG |
| HogA -site-p4 | CATCAGTGCCTCCTCTCAGACAGCTAATGAATCGCGTGGATGC |
| HogA -site-p5 | GCAATGCCGAAGAGTGATGTC |
| HogA -site-p6 | GTTAGGTTCGATCGAGTCTC |
| HogA -L-D-down | GAAACGTCTCCGTCCATCTGAGGATCTTGAATGCGC |
| HogA -L-de-down | CTGGTCGAAACCATCTGAGGATCTTGAATGCGC |
| HogA -R-D-up | CAGATGGACGGAGACGTTTCGACCAGGTACTACCGTG |
| HogA -R-de-up | GATCCTCAGATGGTTTCGACCAGGTACTACCGTG |
| SidB-P1 | CTCGCATTCTACGTGAGTGATG |
| SidB-P2 | GATGAGGGAGAAGAAGACTC |
| SidB-P3 | GGTGAAGAGCATTGTTTGAGGCGATGGCATAAAGCTTCTGAACCC |
| SidB-P4 | CATCAGTGCCTCCTCTCAGACAGCCCTAACTGGATGCCATTAG |
| SidB-P5 | GTGTCGAAACGAGTAGACAG |
| SidB-P6 | GAACAATACTCTGTACAGCACCG |
| SidB-up | ACCCGCTATTCCCAGTTTTG |
| SidB-down | AGTTCTGCCAGGTTTCGTCC |
| Alc-mobA-F | AGCAGCGGCCGATGGCTTCATTCAT |
| Alc-mobA-R | AACCTCTAGATATCCACCAGTCAG |
| PbsB-p1 | TAAAGGGACTAGTCCTGCAGCAGAGACAGTCCTCTTAGTG |
| PbsB-p2 | CAATTCGTTTAAACCTGCAGGGTCGATAGATCGTGATGCT |
| PbsB-p3 | AAGGGCCAATTCGCGGCCGCCCATTTACACGCCTCCGTTC |
| PbsB-p4 | GAATTGAATTTAGCGGCCGCTGGAAGCGAAGTTCCAGGTC |
| PbsB-F | CCTCATCAACTTCCCTTCTCGT |
| PbsB-R | CGCCTTTCTCCCTTAACTGTCT |
| Ribo-F | GTGTAGATTCAGGCACATTG |
| Ribo-R | ATCATTGAATCATATGGCCC |
